# CRISPR-Cas knockout of miR21 reduces glioma growth

**DOI:** 10.1016/j.omto.2022.04.001

**Published:** 2022-04-06

**Authors:** Lisa Nieland, Thomas S. van Solinge, Pike See Cheah, Liza M. Morsett, Joseph El Khoury, Joseph I. Rissman, Benjamin P. Kleinstiver, Marike L.D. Broekman, Xandra O. Breakefield, Erik R. Abels

**Affiliations:** 1 Departments of Neurology and Radiology, Massachusetts General Hospital, Neuroscience Program, Harvard Medical School, Boston, MA 02129, USA; 2 Department of Neurosurgery, Leiden University Medical Center, 2300 RC Leiden, the Netherlands; 3 Department of Human Anatomy, Faculty of Medicine and Health Sciences, University Putra Malaysia, Serdang 43400, Malaysia; 4 Center for Immunology and Inflammatory Diseases, Massachusetts General Hospital and Harvard Medical School, Boston, MA 02129, USA; 5 Center for Genomic Medicine and Department of Pathology, Massachusetts General Hospital, Boston, MA 02115, USA; 6 Department of Pathology, Harvard Medical School, Boston, MA 02114, USA; 7 Department of Neurosurgery, Haaglanden Medical Center, 2512 VA The Hague, the Netherlands; 8 Department of Cell and Chemical Biology, Leiden University Medical Center, 2300 RC Leiden, the Netherlands

**Keywords:** glioblastoma, CRISPR, gene editing, microRNA, miR-21

## Abstract

Non-coding RNAs, including microRNAs (miRNAs), support the progression of glioma. miR-21 is a small, non-coding transcript involved in regulating gene expression in multiple cellular pathways, including the regulation of proliferation. High expression of miR-21 has been shown to be a major driver of glioma growth. Manipulating the expression of miRNAs is a novel strategy in the development of therapeutics in cancer. In this study we aimed to target miR-21. Using CRISPR genome-editing technology, we disrupted the miR-21 coding sequences in glioma cells. Depletion of this miRNA resulted in the upregulation of many downstream miR-21 target mRNAs involved in proliferation. Phenotypically, CRISPR-edited glioma cells showed reduced migration, invasion, and proliferation *in vitro*. In immunocompetent mouse models, miR-21 knockout tumors showed reduced growth resulting in an increased overall survival. In summary, we show that by knocking out a key miRNA in glioma, these cells have decreased proliferation capacity both *in vitro* and *in vivo*. Overall, we identified miR-21 as a potential target for CRISPR-based therapeutics in glioma.

## Graphical abstract

**Figure:**
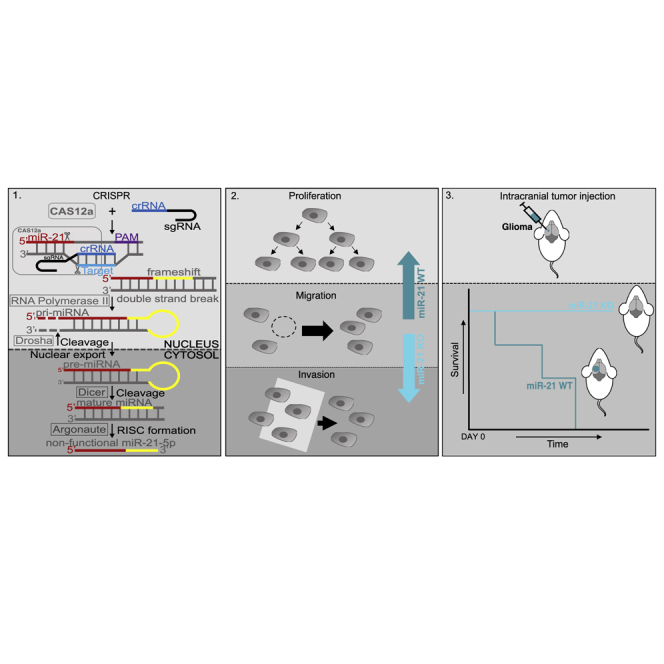

## Teaser

This study presents a gene-editing strategy to knock out miR-21, an oncogene important for tumor progression, highly upregulated in glioma. Glioma cells lacking miR-21 show reduced growth, resulting in increased survival. Targeting this single microRNA shows the potential for CRISPR-based therapeutics in glioma.

## Introduction

Glioblastomas are the most common and lethal primary tumors of the central nervous system. They are known for their extensive cellular heterogeneity, among patients but also within tumors, which makes treatment challenging.^1^ Despite efforts to improve existing treatments, the prognosis of this highly malignant glioma remains poor. In the last few decades there have been no significant improvements in mortality rates, generating an urgency to develop novel treatment strategies.

MicroRNAs (miRNAs) are short, evolutionarily conserved, non-coding RNA molecules; involved in translational repression, targeted cleavage, and deadenylation of messenger RNAs (mRNAs).^2^ Dysregulation of miRNA expression has been observed in many different cancer types, including gliomas.^3^ Interestingly, aberrant expression of miR-21 has been reported in many cancers and is highly upregulated in glioblastoma.^4^, ^5^, ^6^ This miRNA is also involved in embryogenesis, self-renewal, and development, and is among the most studied miRNAs in cancer research.^7^ Murine miR-21 is located on chromosome 11 (human miR-21 is located on chromosome 17) within the intronic region of the gene for vacuole membrane protein 1 (*Vmp1*) (synonym *Tmem49*), a gene involved in autophagy and apoptosis.^8^^,^^9^ Previously, miR-21 has been implicated in the pathogenesis of various cancers, making it a candidate biomarker and a potential target for therapeutics. To investigate the specific role of miR-21 in tumor growth, gain- and loss-of-function assays are frequently used to study which genes are regulated by this miRNA.^10^ Many studies have been able to inhibit or reduce miRNA expression by using antisense oligonucleotide inhibitors or miRNA sponges (sponge RNAs contain complementary binding sites to an miRNA of interest and are produced from transgenes within cells).^11^, ^12^, ^13^ However, the effectiveness of these inhibitors has not been robust, partly due to the short length—∼22 nt—of miRNAs.^14^ A more reliable technique to study the loss of function of miRNA with high efficiency and specificity is establishing a knockout (KO) of the genomic encoding sequences. Therefore, the aim of this study was to target and knock out the miR-21 sequences via clustered regulatory interspaced short palindromic repeats (CRISPR)-Cas technology.

It has recently been described that expression levels of miR-21 in tumors are inversely correlated with the length of survival of glioblastoma patients.^15^ In detail, *SOX2* has been identified as a target of miR-21 in glioblastoma and has led to the characterization of a high-miR-21/low-*SOX2* glioblastoma subtype associated with poor survival outcome.^16^ In addition, elevated miR-21 levels in extracellular vesicles derived from cerebrospinal fluid (CSF) have been used as a biomarker linked to worse prognosis in glioma patients.^17^^,^^18^ Together, these findings suggest that elevated miR-21 levels have an important role in tumor progression and poor survival among glioma patients.

Here, we present a strategy to KO miR-21 expression by targeting the alleles encoding miR-21 using an efficient CRISPR-Cas12a genome-editing system. Overall, we identify miR-21 as a potential target for CRISPR-based therapeutics in glioma.

## Results

### Gene editing resulted in KO of miR21

**Figure 1:**
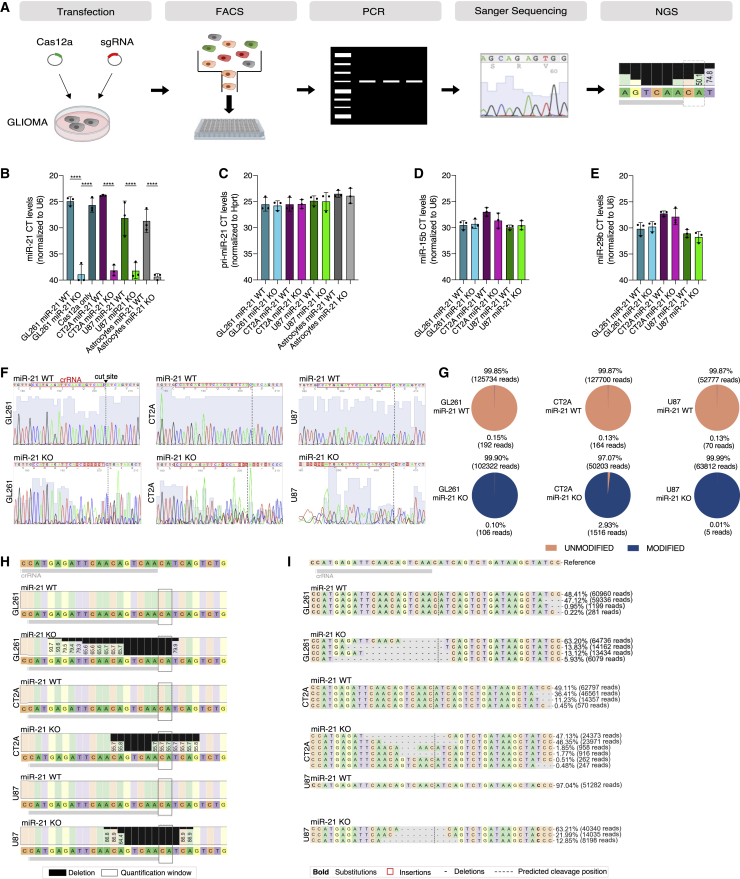
miR-21 KO GL261 and CT2A cell lines generated by CRISPR-Cas12a (A) Schematic overview of the CRISPR approach and analysis to validate the generation of a miR-21 KO GL261 cell line. (B) miR-21 expression levels as determined by TaqMan qRT-PCR. Expression levels of miR-21 normalized to the housekeeping gene U6. CT values showed significant decrease in levels of miR-21 in the CRISPR-edited clones (KO1–3) compared with WT and single transfected cells (Cas12a only). KO CT values were similar compared with CT values of primary astrocytes derived from miR-21 KO mice. Data represent three independent experiments and are presented as the mean with SEM (error bars). Multi-comparison one-way ANOVA, ∗∗∗∗p < 0.0001. (C) Expression levels of pri-miR-21 normalized to the housekeeping *Hprt*. CT values show no significant differences comparing miR-21 KO1–3 and miR-21 WT for GL261, CT2A, U87, and primary astrocytes. Reactions were done in triplicate using the TaqMan assay. Data represent three independent experiments and are presented as the mean with SEM (error bars) (D and E) Two non-targeted miRNAs were tested. Both miR-15b (D) and miR-29b (E) showed no significant changes in expression levels comparing miR-21 KO and the miR-21 WT GL261, CT2A, and U87 cells. Data represent three independent experiments and are presented as the mean with SEM (error bars) (F) Sanger sequencing of PCR products spanning miR-21 using 4Peaks software shows position of target site in miR-21 and reverse sequencing traces of GL261, CT2A, and U87 WT compared with genetically engineered GL261, CT2A, and U87 miR-21 KO clones. The specific crRNA is highlighted in red, and the cut site is displayed with a dotted line. Blue shading behind the peaks represents base quality. WT shows single-base peaks while the miR-21 KO clones show double peaks due to a disruption as a result of the CRISPR edit. (G) NGS was performed, and analysis was conducted using CRISPResso2 to investigate the INDEL formation within a specified quantification window. WT cells showed no modification compared with GL261 miR-21 KO (99.90%), miR-21 CT2A KO (97.07%), and U87 miR-21 KO (99.99%) INDEL modifications in the targeted sequence. (H) Nucleotide percentage quantification was carried out on GL261, CT2A, and U87 miR-21 KO clones of their WT sequence and showed deletions (black boxes) within the region of interest. The quantification window is highlighted with a gray line. (I) The charts represent percentages of reads of individual INDELs within the WT compared with miR-21 KO clones (CRISPResso2 analysis). The sequence reads were aligned to the WT reference allele, and percentages and total reads of INDELs are shown for miR-21 KO GL261, CT2A, and U87 clones. WT showed no INDELs compared with the reference sequence with the sgRNA displayed in gray. miR-21 KO clones show INDELs around the cut side.

To achieve the KO of miR-21 loci, we first tested five different CRISPR RNAs (crRNAs) for efficiency. Sanger sequencing and next-generation sequencing (NGS) were used to validate the CRISPR activity and to verify the miR-21 KO in mouse (GL261and CT2A) and human (U87) glioma lines (Figure 1A). In detail, a CRISPR-Cas genome-editing system was used to KO miR-21 gene expression, as illustrated in Figure S1A. The CRISPR-Cas12a plasmids were designed to co-express a fluorescent marker to track transfection efficiency and identify cells that were potentially edited. Following this approach, we identified cells that expressed both the Cas12 (co-expressing GFP) and crRNA (co-expressing mCherry) by means of fluorescence microscopy (Figure S1B). By sorting cells expressing both fluorescent proteins using fluorescence-activated cell sorting (FACS), we were able to select only cells where both gene-editing components were expressed. Subsequently, effective CRISPR activity was determined by disruption of the miR-21 sequence downstream of the specific crRNA sequence (Figures S1C and S2). Subsequently, cells which had undergone CRISPR editing were single-cell sorted and expanded as clones, with all cells in the clone having the identical editing gene miR-21 KO events. These clones and all subsequent experiments were generated using the crRNA “JIR327,” which showed high efficiency and specificity, determined on the basis of DNA Sanger sequencing (Figure S3). First, the generation of miR-21 KO clones was confirmed by testing the expression levels of miR-21 using qRT-PCR and analyzed for the disrupted genomic sequences using CRISPResso2.^19^ We compared the expression levels of miR-21 in GL261 wild-type (WT) cells with GL261 CRISPR-edited cells, CT2A WT cells compared with CT2A CRISPR-edited cells, and U87 WT compared with U87 CRISPR-edited cells labeled as miR-21 KO. As controls we used primary neonatal astrocytes isolated from C57BL/6 (miR-21^+/+^) mice and miR-21^−/−^ mice.^20^ As expected, miR-21 levels of the miR-21 KO lines (KO1–3) for GL261 and CT2A mouse and U87 human glioma cells were significantly lower than the WT cells from which they were derived and were similar to levels in miR-21 KO astrocytes, being at cycle threshold (CT) values that were considered background (Figure 1B) Expression of miR-21 in GL261 cells was not affected by transfection of the Cas12 expression plasmid alone (Figure 1B). To test whether the transcription of miR-21 was disrupted, we analyzed the expression of the pri-miR-21. We found no significant difference between pri-miR-21 WT and pri-miR-21 KO for GL261, CT2A, U87, and miR-21 KO mouse-derived astrocytes (Figure 1C). We also evaluated expression levels of miRNA miR-29b and miR-15b as controls, whereby expression levels were similar in WT samples compared with KO1–3 for both GL261- and CT2A-derived lines (Figures 1D and 1E). Next, the CRISPR-edited clones were aligned to the GL261 WT and the CT2A WT sequences to analyze the CRISPR-induced insertion-deletion (INDEL) (Figure 1F). No changes were observed outside of the target of interest, as determined by Sanger sequencing of the top five off-targets (Table S1) for the miR-21 GL261 KO cell lines compared with the GL261 miR-21 WT cell line (Figure S4). The CRISPR-edited colonies were analyzed using CRISPResso2 and compared with the unedited cells. We generated in total three miR-21 KO colonies each for GL261, CT2A, and U87 cell lines (Figure S5). The percentages of modified and unmodified reads analyzed in the miR-21 locus showed that the GL261 WT cell line had 192 modified reads (0.15%) while the GL261 miR-21 KO clone 3 had 102,322 (99.90%) modified reads. The CT2A WT cell line had 164 (0.13%) modified reads while the miR-21 KO clone 1 had 50,203 (97.07%) modified reads. The U87 WT cell line had 70 (0.13%) modified reads while the miR-21 KO clone 1 had 63,812 (99.99%) modified reads (Figure 1G). Importantly, NGS showed quantification of nucleotide percentages and deletions that occurred within the region of interest in CRISPR-generated miR-21 KO colonies 1–3 (Figure 1H). INDEL quantification represents percentages of total reads of individual INDELs within miR-21 KO GL261, CT2A, and U87 lines aligned to the WT reference allele. The alleles are aligned to the reference sequence, with miR-21 KO GL261, CT2A, and U87 showing INDELs around the cut side compared with no INDELs in the WT sequence (Figure 1I). In summary, we demonstrated a successful approach to CRISPR editing of the miR-21 genomic locus and validated the generation of complete miR-21 KO mouse (GL261 and CT2A) and human (U87) glioma cells.

### miR-21 KO did not affect Vmp1 expression

The genome-editing system caused an INDEL in the miR-21 sequence in an intron of the *Vmp1* gene. To evaluate whether this affected the functionality of this gene, we first analyzed levels of mRNA expression using primer sets targeting different parts of the *Vmp1* gene (Figure S6A). The qRT-PCR results showed similar expression levels compared with GL261 WT cells, assuming that the sequence of the *Vmp1* gene was not affected by the CRISPR editing (Figure S6B). This was confirmed by both western blot analysis and immunohistochemistry of VMP1 protein expression, which did not show any difference in protein levels or intracellular location between WT and KO cells (Figures S6C–S6E). Together, these results confirm that miR-21 KO has no off-target effects on the Vmp1 gene.

### miR-21 KO resulted in upregulation of miR-21 regulated mRNA targets

**Figure 2:**
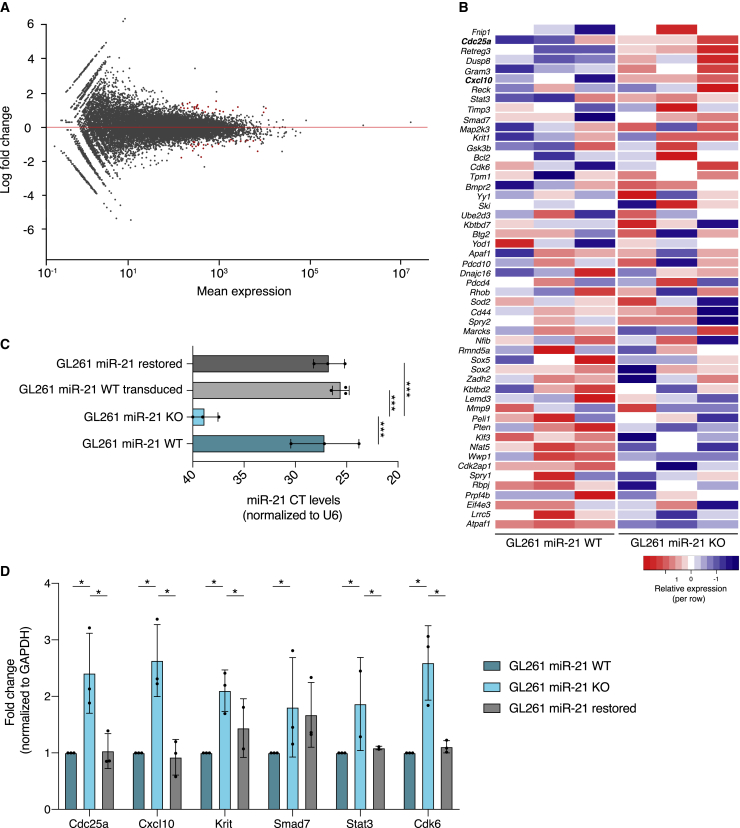
Cells lacking miR-21 showed upregulation of targeted mRNAs (A) MA-plot shows 25 significantly upregulated and 27 downregulated genes (shown in red) when comparing miR-21 KO with miR-21 WT GL261 cells (adjusted p < 0.1). (B) Heatmap shows relative gene expression for 52 validated miR-21 gene targets of the three miR-21 KO clones (KO1–3). (C) miR-21 expression was determined by TaqMan qRT-PCR and normalized to the housekeeping gene U6. CT values were significantly decreased in levels of miR-21 in the CRISPR-edited clones (KO1–3) compared with GL261 WT. CT values for both GL261 miR-21 WT cells and GL261 miR-21 KO cells, transduced with a lentivirus expressing miR-21, were similar compared with the GL261 WT cells. One-way ANOVA, ∗∗∗p < 0.001. Data represent three independent experiments and are presented as the mean with SEM (error bars) (D) Fold expression of miR-21 downstream target genes (*Cdc25a*, *Cxcl10*, *Krit1*, *Smad7*, *Stat3*, and *Cdk6*) were normalized to *Gapdh*. GL261 miR-21 KO-3 and GL261 miR-21 KO-3 with restored miR-21 levels were compared with GL261 miR-21 WT. Data represent three independent experiments and are presented as the mean with SEM (error bars). ∗p < 0.05, unpaired t test and multiple t test.

We performed RNA sequencing (RNA-seq) to compare GL261 miR-21 KO1–3 with miR-21 WT and analyzed the 59 predicted miR-21 mRNA targets in the transcriptome of the GL261 cells. In total, 25 genes were upregulated and 27 genes were downregulated (Table 1) in miR-21 KO cells (significantly changed genes shown in red) compared with miR-21 WT cells (adjusted p < 0.1) (Figure 2A). Next, we evaluated the expression of previously identified targets of murine miR-21.^21^ *Cdc25a* and *Cxcl10* were significantly upregulated (shown in boldface) based on differential expression analysis between miR-21 KO and miR-21 WT GL261 cells (Figure 2B). Next, we restored the level of miR-21 in miR-21 KO cells by stable transduction using a lentiviral vector expressing miR-21 (Figure S7), showing similar levels of miR-21 compared with GL261 WT (Figure 2C). Using further qRT-PCR analysis we validated a number of genes and found that those anti-proliferation genes—cell division cycle 25 homolog A (*Cdc25a*), C-X-C motif chemokine ligand 10 (*Cxcl10*), Krev interaction trapped protein 1 (*Krit1*), signal transducer and activator of transcription 3 (*Smad7*), signal transducer and activator of transcription 3 (*Stat3*), and cyclin-dependent kinase 6 (*Cdk6*)—were differentially expressed to a significant level when comparing miR-21 KO with the cells where we restored miR-21 (Figure 2D). In brief, RNA-seq analysis screening downstream miR-21 targets shows that the lack of miR-21 results in enhanced levels of a number of miR-21 regulated mRNAs. This was validated by using qRT-PCR analysis and by overexpressing miR-21 in miR-21 KO cells, thereby rescuing the levels of miR-21 and restoring the target levels of these mRNAs to equality with the WT cells.

**Table 1:** Significantly differential expressed genes

	Gene	Differential expression comparing GL261 miR-21 KO with WT (log_2_ fold)	Adjusted p value
1	*Slc47a1*	1.52241678139203	0.0041334652355992
2	*Hspb8*	1.44121311601866	0.00484476454212987
3	*Maz*	1.37142775109412	0.0111141937613652
4	*Dbp*	1.35626870544042	0.0317095987372697
5	*D730045B01Rik*	1.3071134852004	0.0109648931209374
6	*I830012 O 16Rik*	1.24483020682272	0.00484476454212987
7	*Abcd1*	1.19528199960586	0.000988306032492133
8	*Sdpr*	1.19273207575752	0.0581221706379791
9	*Lpcat1*	1.18394779874175	0.0325071864396919
10	*Sparcl1*	1.1799963968851	0.0109648931209374
11	*Tnip1*	1.14911265690287	0.0229116722632739
12	*Gm15834*	1.14277945856646	0.00577025620992366
13	*Tef*	1.11406374016995	0.0325071864396919
14	*Atp9a*	1.11065062861786	0.0351929471468051
15	*Lrrc17*	1.10868628941893	0.0498697720215338
16	*Ccne2*	1.09362765856333	0.0351929471468051
16	*Tbc1d10a*	1.06590423746082	0.0109648931209374
18	*Rapgef1*	1.046746578261	0.0041334652355992
19	*Timp2*	0.982246569647121	0.0281426667383727
20	*Mfge8*	0.980387345885256	0.0769306570951875
21	*Rsad2*	0.959747664451954	0.00484476454212987
22	*Ifit1*	0.948322613224547	0.0524427437941683
23	*Col2a1*	0.909510512521087	0.0581221706379791
24	*Ifit3*	0.893156562128124	0.0317095987372697
25	*Sparc*	0.873484368342292	0.0324943156283421
26	*Scand1*	0.840711956338603	0.0823694845089954
27	*Nf2*	0.822028132213232	0.0823694845089954
28	*S100a10*	−0.719290409324988	0.0968296042578051
29	*Rps2*	−0.738218013167964	0.0968296042578051
30	*Rps7*	−0.754652383133823	0.0437344603775375
31	*Tpt1*	−0.779408940562683	0.089397224898311
32	*Rps17*	−0.789821437033326	0.0823694845089954
33	*Rpl13a*	−0.790342277660098	0.0519156556261587
34	*Rps4x*	−0.794549033978309	0.0947735372292341
35	*Rps11*	−0.822735512041855	0.0353516614147008
36	*Bex2*	−0.845757705339494	0.0823694845089954
37	*AI506816*	−0.862389520130167	0.0956669287515029
38	*Flt1*	−0.946975784619228	0.0786395976836765
39	*Tmsb10*	−0.966355536856362	0.0204356152357139
40	*Npm3*	−0.970017122033691	0.0317095987372697
41	*Glud1*	−0.980065393759907	0.0581221706379791
42	*Nhsl1*	−1.01824758815174	0.0947735372292341
43	*U2af1*	−1.03308519008085	0.0823694845089954
44	*Igf1*	−1.04258889006101	0.0745343949374602
45	*Slc1a5*	−1.10588166424938	0.0823694845089954
46	*Pik3r1*	−1.1136310010068	0.089397224898311
47	*Stoml2*	−1.11843628781427	0.0581221706379791
48	*Tdrd7*	−1.22316282159326	0.0351929471468051
49	*Erbb3*	−1.35920200579923	0.0317095987372697
50	*Rpl41*	−1.39187386078865	0.0251520487875858
51	*Tmsb4x*	−1.72519480283298	0.00484476454212987
52	*Gtpbp2*	−1.76138297956782	0.0351929471468051

### Reduced proliferation, migration, and invasion of miR-21 KO cells

**Figure 3:**
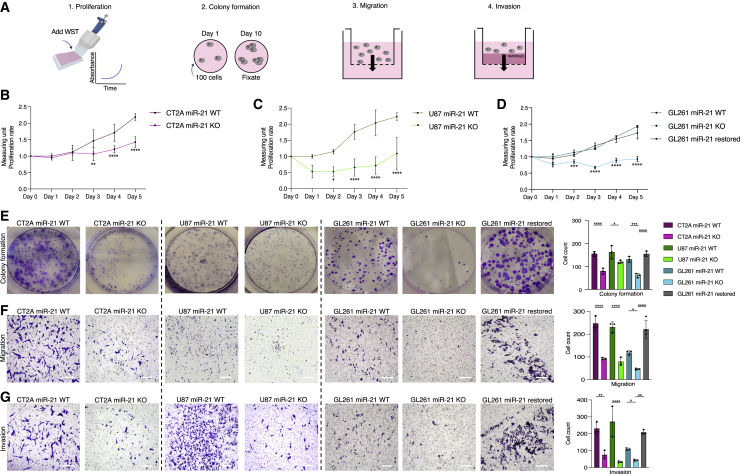
Reduced proliferation, migration, and invasion in miR-21 KO cells (A) Schematic overview of the *in vitro* assays used to study proliferation and colony formation (A1, A2), migration (A3), and invasion (A4). (B) Proliferation rates of CT2A miR-21 WT and KO cells were measured using the WST reduction assay. Cell viability was measured every 24 h for 5 days. The results display the ratio of miR-21 KO compared with WT cells. miR-21 WT cells proliferated at a significantly higher rate compared with the miR-21 KO cells. Data represent triplicates and are presented as the mean with SEM (error bars). ∗∗p < 0.01, ∗∗∗p < 0.001, ∗∗∗∗p < 0.0001, multi-comparison two-way ANOVA. (C) Proliferation rates of U87 miR-21 WT and KO cells were measured using the WST reduction assay. Cell viability was measured every 24 h for 5 days. The results display the ratio of miR-21 KO compared with WT cells. miR-21 WT cells proliferated at a significantly higher rate compared with the miR-21 KO cells. Data represent triplicates and are presented as the mean with SEM (error bars). ∗∗p < 0.01, ∗∗∗p < 0.001, ∗∗∗∗p < 0.0001, multi-comparison two-way ANOVA. (D) Proliferation rates of GL261 miR-21 WT, KO-3, and the KO-3 restored cell were measured using the WST reduction assay. Cell viability was measured every 24 h for 5 days. The results display the ratio of GL261 miR-21 KO and the GL261 miR-21 restored cells compared with GL261 WT cells. WT and restored cells proliferated at a significantly higher rate compared with the miR-21 KO-3 clone. Data represent triplicates and are presented as the mean with SEM (error bars). ∗∗p < 0.01, ∗∗∗p < 0.001, ∗∗∗∗p < 0.0001, multi-comparison two-way ANOVA. (E) Representative images and quantification of colony-formation assay show a significantly lower number of colonies grown of the CT2A, U87, and GL261 miR-21 KO cells and larger colonies for miR-21 restored cells, as compared with the WT cells. miR-21 KO shows a significantly lower number of colonies compared with WT cells. ∗∗∗p < 0.001, ∗∗∗∗p < 0.0001, one-way ANOVA. Data represent three independent experiments and are presented as the mean with SEM (error bars) (F and G) Representative images and quantification of cell migration and invasion measured over 24 h across a transwell membrane with 8 μm pores with (invasion) or without (migration) Matrigel. Both migration (F) and invasion (G) ability of CT2A, U87, and GL261 miR-21 KO cell lines were significantly reduced compared with the miR-21 WT cells. GL261 KO cells were significantly reduced compared with GL261 miR-21 restored cells (∗∗∗∗p < 0.0001, multi-comparison two-way ANOVA). Histograms show the number of cells that grew out as clones and number of cells that crossed the cell membrane. Experiment was repeated three times, and data are presented as the mean with SEM (error bars). ∗∗∗p < 0.001, ∗∗∗∗p < 0.0001, one-way ANOVA.

In many cancers, upregulation of miR-21 is linked to increased cell proliferation.^22^ Therefore, we compared the functional effect of miR-21 KO using *in vitro* proliferation, migration, and invasion assays (Figure 3A). Proliferation rates of GL261, CT2A, and U87 were measured comparing miR-21 WT, KO, and GL261 miR-21-restored cells using the WST-1 reduction assay. Proliferation was significantly decreased in miR-21 KO in both GL261, CT2A, and U87 cells, as compared with miR-21 WT and restored cell lines measured over a 5-day period (Figures 3B–3D). A colony-formation assay was performed to evaluate cell survival based on the ability of single cells to grow into colonies. GL261, CT2A, and U87 miR-21 KO cells produced significantly fewer and smaller colonies compared with the WT cells (Figure 3E). Although the number of the GL261 restored colonies did not differ significantly compared with the GL261 WT colonies, the colony sizes showed enhanced proliferation in cells when the miR-21 was restored. The miR-21-restored cells had significantly more colonies compared with the miR-21 KO cells (Figure 3E, right panel). Interestingly, CT2A cells showed increased migration and invasion over GL261 cells, with a 2-fold higher degree of migration (p < 0.0020) and invasion (p < 0.0043) compared with the GL261. CT2A, U87, and GL621 miR-21 KO cells also showed reduced migration and invasion capacity compared with WT cells, while miR-21-restored GL261 cells (right panel) showed significantly more migrating cells compared with the GL261 miR-21 KO and WT cells (Figures 3F and 3G). In sum, the depletion of miR-21 reduced glioma proliferation, migration, and invasion *in vitro*.

### miR-21 KO showed reduced tumor growth in mice

**Figure 4:**
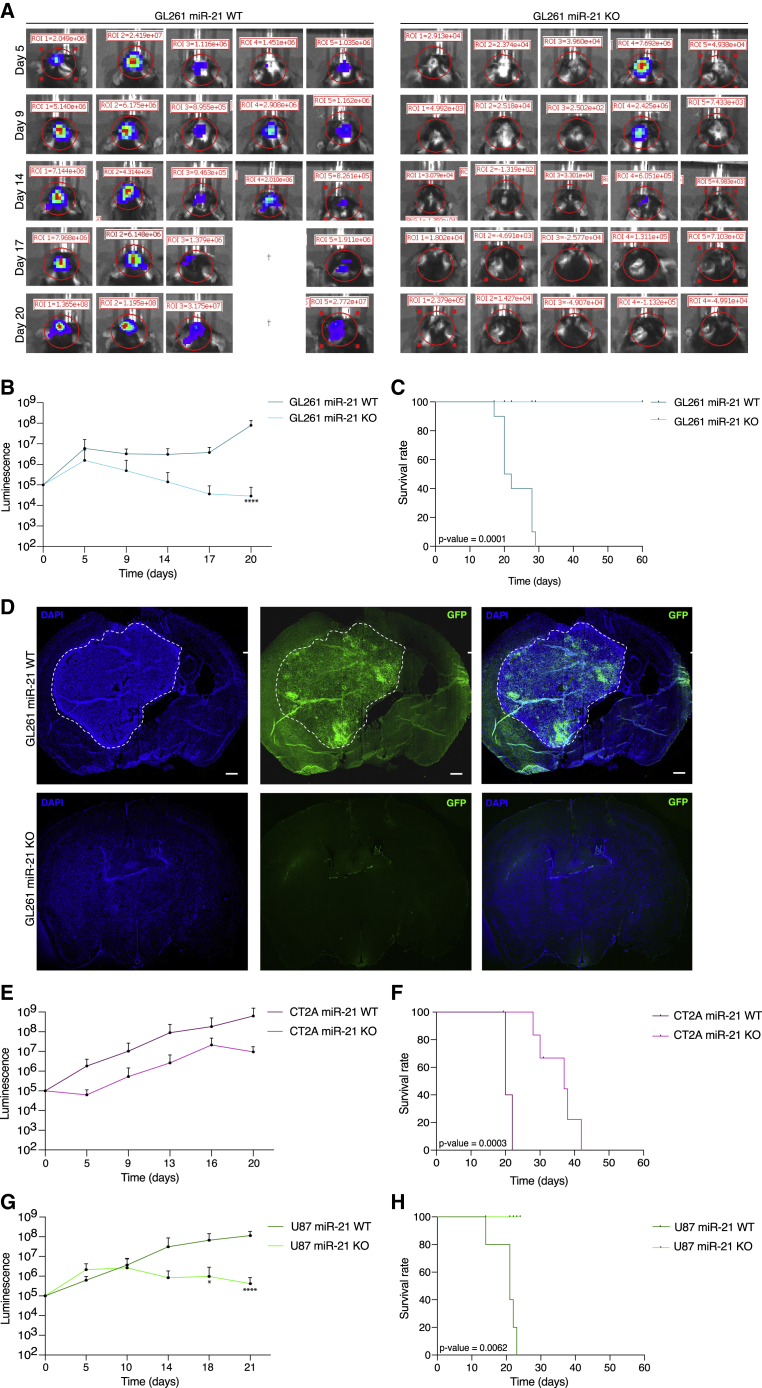
miR-21 KO showed reduced tumor growth in mice (A) Representative IVIS images showing the tumor sizes comparing tumor growth of the GL261 miR-21 WT and GL261 miR-21 KO injected cells at different time points. † indicates animals that died prior to IVIS imaging. (B) Average bioluminescence signal of the mice injected with GL261 miR-21 WT/KO cells over time. GL261 miR-21 KO tumor growth was significantly lower at day 20 (∗∗∗∗p < 0.0001, multi-comparison two-way ANOVA). (C) Kaplan-Meier survival curve after intracranial injection of GL261 miR-21 WT and GL261 miR-21 KO tumor cells. Ten mice injected with GL261 miR-21 WT had a median survival of 21 days. Ten mice injected with GL261 miR-21 KO cells were healthy for up to 60 days, when they were sacrificed for neuropathology. Log-rank test p value = 0.0001. (D) Brain slices were stained with DAPI (blue) and GFP (green) was used to stain the nucleus (the GL261 tumor is marked by a white dashed line). Representative images. Scale bar, 1 mm. (E) Average bioluminescence signal of the mice injected with CT2A miR-21 WT/KO cells over time. Data presented as the mean with SEM (error bars) (F) Kaplan-Meier survival curve after intracranial injection of CT2A miR-21 WT and CT2A miR-21 KO tumor cells. Five mice injected with CT2A miR-21 WT had a median survival of 20 days. Five mice injected with CT2A miR-21 KO cells were healthy for up to 30 days. Log-rank test p value = 0.0090. (G) Average bioluminescence signal of the mice injected with U87 miR-21 WT/KO cells over time. U87 miR-21 KO tumor growth was significantly lower at day 18 and day 21. ∗∗p < 0.01, ∗∗∗∗p < 0.0001, multi-comparison two-way ANOVA. Data presented as the mean with SEM (error bars) (H) Kaplan-Meier survival curve after intracranial injection of U87 miR-21 WT and U87 miR-21 KO tumor cells. Five mice injected with U87 miR-21 WT had a median survival of 21 days. Five mice injected with U87 miR-21 KO cells were healthy for up to 25 days. Log-rank test p value = 0.0062.

To assess the impact of miR-21 KO on overall survival of tumor-bearing mice, we injected GL261, CT2A, and U87 miR-21 KO and WT cells intracranially into the striatum of C57BL/6 mice or BALB/C nude mice. Representative In Vivo Imaging System (IVIS) images of the bioluminescence signal showed reduced tumor sizes in mice injected with the GL261 miR-21 KO cells (Figure 4A). The mice injected with miR-21 KO GL261 cells showed a reduction in tumor growth compared with the mice injected with GL261 miR-21 WT cells, with a significant difference at day 20 (Figure 4B). The miR-21 KO GL261 cell injected mice had a marked increase in survival compared with mice injected with GL261 miR-21 WT cells. Mice harboring the GL261 miR-21 WT tumor showed a median survival of 21 days after tumor cell implantation, whereas GL261 miR-21 KO injected mice lived for at least 60 days in good health (Figure 4C). When GL261 miR-21 KO injected mice were sacrificed, no tumors were observed based on GFP-positive tumor cells compared with GL261 miR-21 WT injected mice (Figure 4D). The mice injected with miR-21 KO CT2A cells showed a reduction in tumor growth compared with the mice injected with CT2A miR-21 WT cells (Figure 4E). The miR-21 KO CT2A cell injected mice had an increase in survival compared with mice injected with CT2A miR-21 WT cells. CT2A miR-21 WT mice died with a median survival of 20 days after tumor cell implantation, whereas CT2A miR-21 KO injected mice lived for at least 30 days in good health (Figure 4F). The mice injected with miR-21 KO U87 cells showed a reduction in tumor growth compared with the mice injected with U87 WT cells, with significant differences at days 18 and 21 (Figure 4G). The miR-21 KO U87 cell injected mice had increased survival compared with mice injected with U87 WT cells. U87 WT mice died with a median survival of 21 days after tumor cell implantation, whereas U87 miR-21 KO injected mice lived for at least 25 days in good health (Figure 4H). To summarize, our data show that miR-21 KO in both mouse and human glioma lines hampered tumor growth and increased overall survival in mice.

## Discussion

We successfully knocked out miR-21 in glioma using the CRISPR technique, as confirmed by non-detectable miR-21 expression levels and NGS. This resulted in decreased proliferation, migration, and invasion of these cells *in vitro*, as well as increased survival of tumor-bearing mice *in vivo*. The cell lines used in this study differed in their growth rate, migration, and invasiveness. The CT2A glioma cell line presents a more aggressive form of glioma. Its higher levels of migration and invasion (as shown in Figure 3) result in faster tumor spread causing the overall survival to be reduced compared with GL261 cells (as shown in Figure 4). Interestingly, even without achieving 100% KO (GL261 KO1: 73%; KO2: 46%; CT2A KO2: 98%; KO3: 44%) based on the genomic integrity of the loci, miR-21 expression levels were markedly reduced, resulting in a functional effect on proliferation in all clones. The pri-miRNA expression did not show differences in levels on comparing miR-21 KO with WT, indicating that the transcription of primary miRNA was not impeded, but its processing and/or maturation to the mature miR-21 was disrupted due to gene editing. Furthermore, our results show that expression of the *Vmp1* gene, in the intron of which miR-21 coding sequences are located, was not changed upon CRISPR editing of the miR-21 genomic sequences, and no off-target activity in sequence-predicted regions of the genome was detected.

Dysregulated expression of miRNAs in cancer cells can act as either a tumor suppressor or oncogene, resulting in inhibition or promotion of tumor development, respectively.^23^ In many cancers, including glioma, miR-21 is highly abundant and functions as an oncogene regulating many different mRNA targets.^24^ Using RNA-seq we screened the miR-21 targets, previously described in the literature, which have been found to be directly regulated by miR-21 in a variety of different cell types.^21^ Using this initial screen, we identified specific miR-21 targets affected by the KO of miR-21 in glioma cells. Here we found several genes that are involved in cell proliferation and apoptosis. First, Cdc25a is overexpressed in a wide variety of cancers and has a crucial role in cell proliferation and apoptosis and, when overexpressed, promotes tumorigenesis.^25^ For example, the tumorigenic effects of Cdc25a are displayed in the transformation of primary mouse embryonic fibroblasts. Here, together with expression of H-RasG12V or in the absence of RB1 these primary mouse embryonic fibroblasts will form tumors.^26^ Counterintuitively, in colon cancer and glioblastoma, Cdc25a upregulation after inhibition of miR-21 resulted in decreased rather than increased tumor proliferation.^27^^,^^28^ In addition, it was shown that Cdc25a has pro-apoptotic properties.^29^ Here, caspase cleavage results in a catalytically active fragment that is localized to the nucleus where it induces apoptosis.^29^^,^^30^ This indicates that the subcellular localization of Cdc25a is an important indicator for its effect on cell fate.^31^ Possibly, the abundance of Cdc25a after miR-21 KO results in nuclear localization and increase in apoptosis in glioma cells.^27^

A similar paradox can be found for Cdk6, which is also involved in cell-cycle regulation. mTOR inhibition and subsequent miR21 upregulation was shown to repress Cdk6 in T cell acute leukemia, resulting in decreased tumor proliferation.^32^ Cdk6 protein levels are elevated in glioma, but this only leads to tumor progression after post-translational modification by SUMOylation, which stabilizes and enhances the kinase activity driving the cell cycle.^33^ Like Cdc25a, the Cdk6 level dictates the miR-21 effect on cells. For example, high levels of Cdk6 have been shown to induce apoptosis rather than proliferation in Kaposi's sarcoma.^34^ Thus, for cell-cycle regulators (Cdc25a and Cdk6) the cellular state or exposure to drugs in combination with how these proteins are post-translationally modified dictates their overall effect on proliferation and apoptosis. In this context it is important to note that prior to targeting miR-21, evaluation is needed as to whether this miRNA acts as a tumor suppressor or an oncogene depending on the mRNA targets that are important within certain cell types or under specific physiological conditions, such as after DNA damage due to irradiation. Also, Krit1 is a gene with a putative role in tumorigenesis, and miR-21 overexpression or silencing Krit1 mRNA levels increases tumor growth.^35^ However, the function of this gene is not well documented, and it is not known whether the miR-21 KO leading to overexpression of Krit1 can tip the scale toward apoptosis, as has been shown for Cdc25a and Cdk6.^29^^,^^34^ More clear-cut is the expression trend and consequences of Cxcl10 increase after miR21 KO. Cxcl10 is reported to be a negative regulator of cell growth in glioblastoma.^36^^,^^37^ Elevated levels of Cxcl10 mRNAs are thus consistent with the observed reduction in miR-21 KO glioma cell proliferation. Moreover, Smad7 is a key regulator of the transforming growth factor β1, which suppresses the migration and invasion capacity of glioma cells.^38^ Thus, increased levels of Smad7 mRNA can reduce the capacity of miR-21 KO cells to migrate. In addition, Stat3, a gene involved in cell proliferation and invasion, is activated in multiple cancers,^39^ including glioma. It was shown that Stat3 and miR-21 closely interact,^40^ and the complex interplay between non-coding RNAs (including miR-21) and Stat3 related to its function in glioma has recently been thoroughly reviewed.^41^ Moreover, miR-21 downregulates human telomerase reverse transcriptase expression through its action on Stat3, inhibiting glioma cell growth.^42^ Therefore, glioma cells lacking miR-21 might upregulate the Stat3 pathways involved in tumor progression. The KO of miR-21 resulting in elevation of mRNAs important in controlling the cell-cycle machinery can cause cell-cycle arrest and promotes apoptosis, together resulting in reduced cell proliferation observed in the miR-21 KO glioma cells.

The discrepancy between miR-21-KO-induced upregulation of genes known to be involved in tumorigenesis and tumor growth inhibition may reflect the ability of miRNAs to buffer the effect of variations in gene expression.^43^ In glioma miR-21 levels are overexpressed, causing downregulation of mRNAs associated with controlling cell proliferation. However, in our miR-21 KO glioma models the feedback networks of miR-21 to regulate downstream mRNAs are lacking completely; this appears to collectively destabilize gene expression that, in the context of glioma, leads to decreased proliferation and invasion, as we observed in the subset of miR-21 targets we analyzed in this study. However, we should note that miR-21 dynamically regulates a network of multiple targets, many of them involved in tumor progression or apoptosis, and in our study we focused only on a subset of targets that might not represent the complete function of miR-21. Therefore, future studies will be needed to better understand the downstream pathways and mechanisms responsible for hampered tumor growth caused by miR-21 KO.

While the expression of Sox2 was previously shown to be affected by miR-21, in our gene-expression analysis using RNA-seq and qRT-PCR (data not shown) we did not find any significant changes in Sox2 mRNA. This was surprising, since this gene has been found to be targeted by miR-21 in other cell types, i.e., human mesenchymal cells,^44^ neural crest stem cells,^45^ and embryonic stem cells.^46^ A possible explanation is that Sox2 is so highly expressed in glioma^47^^,^^48^ and that miR-21 knockdown does not lower levels substantially. In our dataset, Sox2 is expressed in the top 500 (406 of 18,491) genes in GL261. Thus, the absence of miR-21 might not have a marked effect on this level of overexpression.

In prior studies, miR-21 was inhibited by transfection with either 2V-O-methyl-miR-21 or LNA/DNA-miR-21 to elucidate the functional target mRNAs of miR-21.^4^^,^^49^ Inhibiting miR-21 has been shown to be efficient in reducing tumor size and eventually inhibiting tumor progression in multiple cancer types, including breast cancer,^50^ prostate cancer,^51^ and pancreatic cancer.^52^ However, the binding affinity between miRNA and those inhibitors was relatively weak and limited to short-term studies due to irreproducibility after transfection. The use of sponges seemed to overcome this pitfall by showing long-term stability; however, the miRNA sponge was found to be vulnerable to Ago2-mediated endonucleolytic cleavage, which decreased its efficiency.^14^ The CRISPR system has been proved to be a successful genomic tool to KO specific miRNAs *in vitro* and *in vivo* to understand the function of specific miRNAs (i.e., miR-2188, miR-137, miR-182) in multiple different cancer types, including carcinoma, leukemia, and ovarian cancer.^14^^,^^53^, ^54^, ^55^, ^56^ Others have used the CRISPR approach to target miR-21 in various human cell lines such as HEK293, colon cancer HCT-116, prostate cancer LNCaP, and breast cancer MCF-7 cell lines,^57^ and in human colon cancer cell lines RKO and DLD1.^28^ More recently, four different lentiviral vectors containing CRISPR components were used to target the pre-miR-21 hairpin structure at the DNA level, inducing several mutations in ovarian cancer cell lines. This resulted in upregulation of target mRNAs, including those for programmed cell death protein 4 and Sprouty RTK signaling antagonist 2, leading to reduced proliferation as well as increased migration and invasion.^58^

The potential of miR-21 as a promising therapeutic candidate in various types of cancer has been vigorously studied and extensively reviewed.^59^ Encouragingly, an interventional clinical trial is currently being conducted to evaluate six miRNAs (including miR-21) to define high- and low-risk colon cancer patients and determine which patients should receive adjuvant chemotherapy (ClinicalTrials.gov: NCT02466113). Moreover, a clinical trial aimed at suppressing miR-21 in patients with Alport syndrome is under way, where RG-012, a chemically modified oligonucleotide that can bind to miR-21, is administered (ClinicalTrials.gov: NCT03373786). Although anti-miR-21 is an appealing cancer therapy, including for glioma,^15^ further studies are needed to validate its success in large-scale studies. Therefore, a complete understanding of the complex mechanisms regulating the interaction between this miRNA and its targets is needed. Finally, other issues faced by miRNA therapeutics are the potential off-target toxicity and long-term effects. Optimistically the approach presented here, which is based on gene editing, avoids the side effects of current available strategies to inhibit miR-21 (e.g., antisense oligonucleotide inhibitors, locked nucleic acid-modified small interfering RNAs, and miRNA sponges) and should make it possible to safely and effectively target miR-21 in tumor cells *in vivo* for sustained effect.

It is collectively known that elevated miR-21 is involved in tumor progression. As extensively reviewed, miRNA inhibitors are increasingly being implemented in clinical trials.^60^ However, current strategies to inhibit miR-21, such as antisense oligonucleotides,^61^ in combination with S-TRAIL,^62^ are limited due to their side effects and short half-life.^63^ In our study, we used CRISPR technology so as not only to suppress or temporarily knock down the function of miR-21 but to completely remove miR-21 from glioma cells. These established miR-21 KO glioma cell lines allow further elucidation of the function of miR-21 in the context of glioma and also its effect on the tumor microenvironment. Now that we show the striking effect of loss of miR-21 in glioma cells on tumor progression, the next goal should be to focus on how to safely and efficiently deliver the CRISPR constructs, specifically targeting miR-21 selectively at the tumor site. We hypothesize that this could be done with adeno-associated virus (AAV) gene delivery, using AAV capsid variants which preferentially transduce glioma cells,^64^^,^^65^ although other carriers (such as lentiviral vectors) can also be used to deliver the Cas protein and the single guide RNA (sgRNA) to the tumor.^66^

In this study, we show that effective CRISPR KO targeting of miR-21 in mouse gliomas results in reduction of tumor growth. We identified miR-21 as an important target for miRNA-based therapeutics in glioma. Our study paves the way for the advancement of RNA-based therapy in glioma through targeting oncogenic miRNAs. Future studies could apply our approach to other cancers and other miRNAs that are correlated with tumor progression in the context of glioma or other tumor types, such as elevated miR-10b.^67^ Although miR-21-based biomarkers and therapeutics seem very plausible for future use, further large-scale validation studies will be necessary to reveal the clinical significance of miR-21 targeting, and further research is needed to focus on the delivery of these CRISPR therapeutics in combination with other treatment regimens.

## Materials and methods

### Contact for reagent and resource sharing

Further information and requests for resources and reagents should be directed to and will be fulfilled by the lead contact, Erik Abels (e.r.abels@lumc.nl).

### Experimental model and subject details

#### Animals

All animal experiments were conducted under the oversight of the Massachusetts General Hospital Institution Animal Care and Use Committee (IACUC). C57BL/6J, B6;129S6-Mir21atm1Yoli/J (miR-21^−/−^) mice^20^ and CAnN.Cg-Foxn1nu/Crl (BALB/c) nude mice from Charles River Labs (IACUC protocol 2009N000054) were maintained with unlimited access to water and food under a 12-h/12-h light/dark cycle. To study tumor proliferation *in vivo*, we used a total of 20 C57BL/6J adult male mice randomly assigned to each group.

#### Cell culture

The National Cancer Institute (NCI) provided glioma cells (GL261, CT2A, and U87). All cells were cultured at 37°C in a 5% CO_2_ humidified incubator. GL261 cells were cultured in medium containing Roswell Park Memorial Institute (RPMI) 1640 with L-glutamine (Corning, Glendale, AZ). CT2A and U87 cells were cultured in Dulbecco’s modified Eagle’s medium (DMEM; Corning) supplemented with penicillin (100 units/mL)-streptomycin (100 μg/mL) (P/S) (Corning) and 10% fetal bovine serum (FBS) (Gemini Bioproducts, West Sacramento, CA). Cells were tested negative for mycoplasma contamination at periodic intervals throughout the study (Mycoplasma PCR Detection Kit G238; ABM, Richmond, BC, Canada).

For *in vivo* experiments, miR-21 WT and KO (GL261, CT2A, and U87) cells were stably transduced with a lentiviral vector to co-express FLuc and GFP separated by an internal ribosome entry site element. Following transduction, cells were sorted based on the expression of GFP. Cells with stable expression of GFP and FLuc (GL261.miR21-WT/KO.FLucGFP cells, CT2A.miR21-WT/KO.FLucGFP cells, and U87.miR21-WT/KO.FLucGFP cells) were used for all subsequent *in vivo* experiments.

For the downstream mRNA expression analysis, both GL261 WT and GL261 miR-21 KO cells were stably transduced with a lentivirus vector to co-express the inserted miR-21 sequence and GFP under a cytomegalovirus (CMV) promoter (LentimiRa-GFP-mmu-mir-21 vector, mm10221; ABM). A schematic vector map is shown in Figure S7. Following transduction, cells were sorted based on the expression of GFP. miR-21 expression levels were measured using the TaqMan assay (000397; Applied Biosystems, Beverly, MA) to determine successful transduction.

### Method details

#### Intracranial tumor injection

Adult mice were anesthetized using 2.5% isoflurane in 100% oxygen via a nose cone. A total of 1 × 10^5^ GL261.miR-21-WT, CT2A.miR-21-WT, U87.miR-21-WT, or miR-21 KO FlucGFP cells was suspended in 2 μL of Opti-MEM (Gibco, Waltham, MA). The cells were then implanted into the left striatum of 20 C57BL/6J and 10 BALB/c nude mice using a Hamilton syringe (Sigma-Aldrich, Germany) and automatic stereotaxic injector (Stoelting, Wood Dale, IL) with a flow rate of 0.2 μL/min. In reference to bregma, three coordinates for stereotactic implantation were chosen: anterior-posterior (AP) = 2.0 mm, medial-lateral (ML) = −0.5 mm, and dorsal-ventral (DV) = −2.5 mm. Overall survival of the mice was based on 20% weight loss or if they were under clear distress and their actual death. Tumor growth in mice was followed by measuring bioluminescence using IVIS (PerkinElmer, Waltham, MA).

#### CRISPR-Cas12a plasmids

Five different plasmids (Table 2) encoding different guide RNAs (gRNAs) expressed under a U6 promoter were tested. Each contained a specific crRNA aligned to a different part of the miR-21 sequence (Figure S2). These gRNAs were named JIR320, JIR321, JIR324, JIR327, and JIR328, and co-expressed with mCherry under a separate U6 promoter. Protospacer adjacent motifs (PAMs) and crRNA sequences are shown in Table 2. They were used in combination with a plasmid expressing Cas12a under a CMV promoter, pCMV-T7-enAsCas12a-NLS (nuc)-3xHA-P2A-EGFP (RTW2896).

**Table 2:** CRISPR plasmids

	Plasmid name	Plasmid ID	PAM	crRNA sequence
1	pUC19-U6-AsCas12a_crRNA-miR-21-site1	JIR320	CTTG	5-TCGGATAGCTTATCAGACTG-3′
2	pUC19-U6-AsCas12a_crRNA-miR-21-site2	JIR321	CTTA	5-TCAGACTGATGTTGACTGTT-3′
3	pUC19-U6-AsCas12a_crRNA-miR-21-site5	JIR324	GTTG	5-AATCTCATGGCAACAGCAGT-3′
4	pUC19-U6-AsCas12a_crRNA-miR-21-site3	JIR327	GTTG	5-CCATGAGATTCAACAGTCAA-3′
5	pUC19-U6-AsCas12a_crRNA-miR-21-site4	JIR328	GTTG	5-ACTGTTGAATCTCATGGCAA-3′

#### Transfection

Glioma (GL261, CT2A, and U87) cells were transfected using Lipofectamine 2000 (Thermo Fisher Scientific, Cambridge, MA) in Opti-MEM using the manufacturer’s protocol. In detail, 1 × 10^6^ glioma cells were plated in each well of 6-well plates (Corning) and transfected with plasmid DNA containing the CRISPR plasmids: 1 μL of Cas12a (RTW2896) (5 μg/μL) and 1 μL of pUC19-U6-AsCas12a_crRNA-miR-21-site3 (JIR327) (5 μg/μL). Transfection mixes were incubated at room temperature for 30 min before adding to the cells. Cells were subsequently incubated for 6 h with the transfection mix at 37°C. Transfection efficiency was monitored by level of GFP and mCherry fluorescence after 24 h, with corresponding microscopy images acquired on a Zeiss Axio Imager M2 (Carl Zeiss, Peabody, MA).

#### Fluorescence-activated cell sorting

First, to test the editing efficiency of the different sgRNAs, cells were sorted in bulk after transfection. Cells transfected with different plasmids were stained with 4′,6-diamidino-2-phenylindole (DAPI; Invitrogen, Waltham, MA) at final concentration of 1 μg/mL. Live cells were sorted by selecting only DAPI-negative cells using a BD FACSAria II SORP Cell Sorter (Biosciences, Woburn, MA). Glioma cells co-transfected with GFP (CAS12a plasmid) and mCherry (sgRNA plasmid) were sorted by FACS. Gates were set to only sort cells positive for both GFP and mCherry. Live cells were plated as single cells in 96-well plates containing 100 μL of complete culture medium to select single clones. Collected cells were grown over a period of 2–3 weeks. After expanding the colonies, different aliquots were frozen in RPMI medium with 20% FBS and 10% DMSO and stored at −80°C.

#### Primary astrocyte cultures

Mixed glial cultures were isolated from cerebral cortices of P1 to P4 C57BL/6J or miR-21 KO mouse pups.^20^ Meninges were removed, and cortical cells were dissociated using 0.05% trypsin/EDTA (Corning) followed by single-cell suspension preparation using 100 mm and 40 mm cell strainers (BD Falcon). Cells were cultured in DMEM with 20% FBS, 1% P/S, and 10 ng/mL M-CSF (Gibco, Waltham, MA) on poly-D-lysine (PDL; Sigma-Aldrich, Germany; 10 mg/mL) pre-coated T-75 culture flasks for 10–15 days. Primary microglia were removed from confluent mixed glial culture by gentle shaking on an orbital shaker for 1 h at 180 rpm. Astrocytes were collected in the medium by centrifugation 300 × *g* after further shaking overnight at 230 rpm.

#### DNA sequencing

Genomic DNA (gDNA) was extracted using Quick extract (Lucigen, Middleton, WI) and quantified using a NanoDrop (Wilmington, DE) ND-1000. First, the miR-21 alleles were amplified, using the Phusion High Fidelity PCR kit (New England Biolabs, Ipswich, MA), with two different primer sets specifically designed to flank the target sequences. Primers, forward (5′-GGT TCA CCT AGA GTG GGA ATC T-3′) and reverse (5′-ATT GGG GTA GTC GTC ACA GTC-3′) amplifying 625 bp DNA fragment where miR-21 (mouse) is encoded; and primers, forward (5′-CAT CGT GAC ATC TCC ATG GCT-3′) and reverse (5′-ACC ACG ACT AGA GGC TGA CTT-3′) amplifying 506 bp DNA fragment where miR-21 (human) is encoded were specifically designed for Sanger DNA sequencing analysis. The primers, forward (5′-TTG ACT GCA AAC CAT GAT GCT G-3′) and reverse (5′-TGC TTT AAA CCC TGC CTG AGC-3′) encoding miR-21 (mouse); and primers, forward (5′-TCG TGA CAT CTC CAT GGC TG-3′) and reverse (5′-GTG CCA CCA GAC AGA AGG AC-3′) encoding miR-21 (human) 205 bp were specifically designed for NGS analysis. For off-target analysis, five primer sets were used (Table S1). All primers were developed using primer-blast (http://www.ncbi.nlm.nih.gov/tools/primer-blast). After amplification, the PCR products were purified using the Qiagen PCR purification kit. Next, the PCR products were loaded on a 2% agarose gel (Bioresearch, Worcester, MA), and DNA fragments were resolved by gel electrophoresis. In parallel, the purified PCR products were sequenced using the forward primer 5′-TTG ACT GCA AAC CAT GAT GCT G-3′. To confirm CRISPR activity, we used both Sanger sequencing and NGS analysis, and determined CRISPR efficiency based on CRISPResso2 analysis by using the online tool http://crispresso2.pinellolab.org.^19^

#### qRT-PCR

Total RNA was extracted using the Direct-Zol MicroRNA kit (Zymo-research, Irvine, CA). For gene-expression analysis using qRT-PCR, cDNA was synthesized from 200 ng of total RNA and prepared using the SuperScript VILO cDNA Synthesis kit (Invitrogen). qRT-PCR analyses were performed using SYBR green (for *Vmp1* mRNA expression analysis) (Table 4) or TaqMan (for (pri)-miRNA and miR-21 downstream mRNA expression analysis). Gene expression was determined using the SYBR green protocol qPCR mix, as prepared following the manufacturing protocol of Power SYBR Green PCR Master Mix (Applied Biosystems, Beverly, MA). The cycling conditions using the fast protocol were 50°C for 2 min, 92°C for 10 min, and 40 cycles of 95°C for 1 s and 60°C for 20 s. The *Vmp1* expression was normalized to the housekeeping gene *β-Actin*.

miRNA levels were analyzed using the TaqMan microRNA assay (Table 3) following manufacturing protocol. First, cDNA was synthesized using a TaqMan MicroRNA Reverse Transcription Kit (Applied Biosystems) in combination with miR-21-specific (000397; Applied Biosystems) stem-loop reverse transcription primers and the housekeeping gene U6 (001973; Applied Biosystems). Cycling conditions used in qPCR were 95°C for 20 s, 40 cycles at 95°C for 1 s, and 60°C for 20 s. Pri-miRNA expression was tested using TaqMan chemistry. cDNA synthesized, as previously described, was used together with primers for pri-miR-21 (mm_03306822_pri; Applied Biosystems) and primers specific for *Hprt1* (mm00446968; Applied Biosystems) as a housekeeping gene. The cycling conditions used were 50°C for 2 min, followed by 2 min at 95°C, and 40 cycles of 95°C for 1 s and 60°C for 20 s. The qPCR mixes were prepared with TaqMan Fast Advanced Master Mix (Applied Biosystems) following manufacturing protocol and performed in triplicate. All qPCR reactions were performed using the QuantStudio 3 PCR system (Applied Biosystems).

**Table 3:** TaqMan primers

Primers	Assay ID and manufacturer
U6	001973, Applied Biosystems
miR-21	000397, Applied Biosystems
pri-miR-21	Mm_03306822_pri, Applied Biosystems
miR-15b	000390, Applied Biosystems
miR-29b	000413, Applied Biosystems

mRNA expression levels of downstream miR-21 targets *Cdc25a* (Mm00483162_m1), *Cxcl10* (Mm00445235_m1), *Krit1* (Mm01316552_m1), *Smad7* (Mm00484742_m1), *Stat3* (Mm01219775_m1), and *Cdk6* (Mm01311342_m1) were analyzed using the TaqMan protocol. cDNA was synthesized from 400 ng of total RNA and prepared using the SuperScript VILO cDNA Synthesis kit (Invitrogen). The qPCR mixes were prepared with TaqMan Fast Advanced Master Mix (Applied Biosystems) following manufacturing protocol and performed in triplicate.

All qPCR reactions were performed using the QuantStudio 3 PCR system (Applied Biosystems) and normalized to the housekeeping gene *Gapdh* (Mm99999915_g1). The pri-miRNA expression was normalized to the housekeeping gene *Hprt1* (Mm00446968). The *Vmp1* expression was normalized to the housekeeping gene *β-Actin*.

#### Primers

Eleven sets of mmu-*Vmp1* primers (Table 4) were developed using primer-blast (http://www.ncbi.nlm.nih.gov/tools/primer-blast) to specifically target the exons of the *Vmp1* gene by qRT-PCR as listed below.

**Table 4:** Eleven sets of mmu-*Vmp1* primers

Primer number	Primer sequence	Amplicon location within *VMP1* mRNA
Fw1	5-CCAGAGACGCATAGCAATGAG-3′	27–48
Rv1	5-GCAAGGTAATGAGTGGCTGTC-3′	142–163
Fw2	5-AGCCACTCATTACCTTGCAGT-3′	146–167
Rv2	5-TGCCACAATTTTGAGGTCCATT-3′	146–221
Fw3	5-GGACCTCAAAATTGTGG CATCG-3′	203–225
Rv3	5-CGCTGCACATACTGTTGGTG-3′	294–314
Fw4	5-TGCAGCGGATAGAGAAGCAG-3′	431–450
Rv4	5-TGTGGGCCCAGATAAAGCAG-3′	526–545
Fw5	5-ATTGAAGCCTGCATGTGGGG-3′	681–700
Rv5	5-GCAAAGTCTTGTGCAGCCTC-3′	819–838
Fw6	5-CAAGACTTTGCATCACGGGC-3′	828–847
Rv6	5-AGGTCAAACAGGGGGTTTGG-3′	915–934
Fw7	5-GCAACCCTGATTGGGAAAGC-3′	978–1006
Rv7	5-AAGTCACCATCTGCTCCACG-3′	1064–1083
Fw8	5-CCGTCTCTGCAGAAGCCTTT-3′	1110–1129
Rv8	5-GCCCGCTTCACTTCTGTGAT-3′	1165–1184
Fw9	5-CTGGTGGTTGCAATGGTGTG-3′	1227–1246
Rv9	5-GTTCAAGCGCTGCTGGATTC-3′	1297–1316
Fw10	5-TAAGCCCCAGCAAACCAGAG-3′	2394–2413
Rv10	5-ATCCGACAAGGTGGTACAGC-3′	2485–2504
Fw11	5-TACCCAGCATCATGGTTTGC-3′	2546–2627
Rv11	5-GGTATAAGGGCTCC AAGTCTCA-3′	2575–2696

#### Western blot

Total protein was extracted from the cells using RIPA lysis buffer with presence of protease inhibitor cocktail (Sigma-Aldrich). Protein concentration was determined using the Pierce BCA protein assay (Thermo Fisher Scientific). Equal amounts of protein (20 μg) were loaded and resolved on 10% SDS-PAGE gels (Thermo Fisher Scientific). After transfer onto nitrocellulose membranes, samples were probed with primary antibodies for TMEM49/VMP1 (D1Y3E, 1:1,000; Cell Signaling, Danvers, MA), GAPDH (CB1001, 1:1,000; Millipore, Danvers, MA). Secondary antibodies were ECL anti-rabbit immunoglobulin G (IgG) (Sigma-Aldrich) and ECL anti-mouse IgG (Thermo Fisher Scientific) (1:5,000) corresponding to the primary antibody.

#### Immunofluorescence staining

For imaging, cells were seeded on PDL-coated glass coverslips and incubated for 48 h prior to transfection. Transfection efficiency was analyzed 24 h post transfection. After transfection, cells were rinsed in PBS for 5 min and fixed using 100% ice-cold methanol for 10 min. After fixation, cells were rinsed twice in PBS for 5 min each. Blocking was achieved by using 5% BSA and 0.1% Tween 20 in PBS (PBS-T) for 4 h. Cells were then incubated with the primary antibody TMEM49/VMP1 anti-rabbit (D1Y3E, 1:400; Cell Signaling) at 4°C overnight. Cells were rinsed three times in PBS-T for 5 min each. Secondary antibody goat anti-rabbit (1:400) (Invitrogen) was diluted in PBS-T and incubated for 1 h in the dark at room temperature. Cells were counterstained for DAPI (1 mg/mL) (Thermo Fisher Scientific) diluted in PBS-T and incubated for 10 min at room temperature. Coverslips were transferred to microscope slides (Fisherbrand, Ottawa, ON, Canada) on a droplet of mounting medium (Vectashield; Vector Labs, San Francisco, CA). Fluorescence microscopy images were acquired on the Zeiss Axio Imager M2 (Carl Zeiss) and were processed using ImageJ 1.49v software.

Brain slices on microscope slides (Fisherbrand) were fixed with 4% paraformaldehyde (PFA) for 10 min at room temperature. After fixation, the slices were rinsed with PBS for 5 min and blocked by using 5% BSA and 0.1% PBS-T for 1 h at room temperature. Brain slices were then incubated with the primary antibody GFP anti-mouse (1:400) (Invitrogen) at 4°C overnight. Cells were rinsed three times in PBS-T for 5 min each. Secondary antibody goat anti-mouse (1:400) (Invitrogen) was diluted in PBS-T and incubated for 1 h in the dark at room temperature. Slides were then mounted with DAPI (Vectashield; Vector Labs). Fluorescence microscopy images were acquired on a Keyence (Itasca, IL) microscope and processed using ImageJ 1.49v software.

#### RNA sequencing

The RNA concentration and integrity (RIN score) were determined using the Agilent 2100 Bioanalyzer Pico-chips (Agilent Technologies, Lexington, MA) according to the manufacturing protocol. RNA libraries were prepared from the extracted RNA using QuantSeq 3′ mRNA-seq library prep kit for Illumina (Lexogen, Vienna, Austria). Library amplification and library barcoding were performed using the i7 Index Plate for QuantSeq for Illumina barcodes 7001–7096 (Lexogen), and 13 cycles of library amplification were completed. Equal molar individual libraries were pooled, and the pool concentration was determined using the SYBR FAST Universal qPCR Kit. Finally, libraries were diluted and denatured with the addition of 1% PhiX Sequencing Control V3 (Illumina, San Diego, CA). Single reads were generated using MiniSeq High Output kits (75 cycles) on a MiniSeq (Illumina).

#### Cell proliferation assay

Cell proliferation was assessed *in vitro* by the WST reduction assay to determine cell viability (cell counting kit-8 [CCK-8]; Dojindo, Rockville, MD). Cells were seeded at a low density (2 × 10^3^ cells/well) in a 96-well plate. After 24 h the medium was changed, and 10% WST solution was added to the cells. The cells were incubated at 37°C for 1 h, and fluorescence at wavelength 450 nm was measured using a microplate reader (SynergyH1; BioTek, Winooski, VT). Thereafter, the medium was changed and the cells were measured again 24 h later up until 80% confluency at day 3.

#### Cell migration and invasion

Cell migration and invasion assays^68^ were performed *in vitro* using Boyden chamber transwell membranes with a permeable polycarbonate membrane containing 8 μm pores separating the two chambers (Corning). For the invasion assay, the membranes were first coated with 500 μL of Matrigel (Corning) diluted in serum-free medium (1:5) at 37°C for 1 h. One milliliter of migration buffer (medium containing 10% FBS) was added to the lower chamber and incubated for 30 min at 37°C. A total of 1.8 × 10^4^ cells was plated in the upper chamber in 0.5% FBS and incubated for 24 h at 37°C. After 24 h the transwell was removed from the plate, 750 μL of 70% ethanol was added per well in a 24-well plate, and the transwell was placed into the well to fixate the cells which had migrated through the membrane and attached to the transwell. The transwell was removed and dried for 10 min. Next, 750 μL of 0.2% crystal violet (Sigma-Aldrich) was added to the well and incubated for 30 min. Migrated cells were imaged under an inverted microscope and counted using ImageJ.

#### Colony formation

Colony-formation assay was performed as described previously.^69^ Cells were plated at low density (∼100 cells per well) in 6-well plates and allowed to grow into colonies for 10–14 days. Cells were then fixed for 5 min in 4% PFA and stained with 0.05% crystal violet (Sigma-Aldrich) for 30 min. Cells were washed twice with PBS and imaged to count the colonies by ImageJ.

#### Data processing and analysis

Raw sequencing data were processed and aligned with the Bluebee platform using the Lexogen QuantSeq 2.3.6 FWD data analysis workflow. Data were aligned to mouse genome GRCm38. After aligning and read counting, differential expression analysis was performed with DESeq2 (version 1.30.1)^70^ in R (version 4.0.5). Differential expression analysis, as performed in DESeq2, was subjected to statistical significance using Benjamini and Hochberg multiple testing adjusted p values, with an adjusted p value of <0.05 labeled as significant. Regularized logarithm (rlog) values were used for unsupervised clustering of the most differential expressed genes between samples, and heatmaps were plotted using the pheatmap package (version 1.0.12) in R using scripts previously described^71^. Raw and processed transcriptomic data described in this paper are deposited in NCBI Gene Expression Omnibus (GEO) and are accessible using GEO series accession number GEO: GSE182390.

## Supplemental information

Document S1. Figures S1–S7; Tables S1 and S2

Document S2. Article plus supplemental information

## References

[1] Inda M.M., Bonavia R., Seoane J. (2014). Glioblastoma multiforme: a look inside its heterogeneous nature. Cancers (Basel).

[2] Winter J., Jung S., Keller S., Gregory R.I., Diederichs S. (2009). Many roads to maturity: microRNA biogenesis pathways and their regulation. Nat. Cell Biol.

[3] Ahmed S.P., Castresana J.S., Shahi M.H. (2021). Glioblastoma and miRNAs. Cancers.

[4] Chan J.A., Krichevsky A.M., Kosik K.S. (2005). MicroRNA-21 is an antiapoptotic factor in human glioblastoma cells. Cancer Res..

[5] Moller H.G., Rasmussen A.P., Andersen H.H., Johnsen K.B., Henriksen M., Duroux M. (2013). A systematic review of microRNA in glioblastoma multiforme: micro-modulators in the mesenchymal mode of migration and invasion. Mol. Neurobiol..

[6] Sheedy F.J. (2015). Turning 21: induction of miR-21 as a key switch in the inflammatory response. Front. Immunol..

[7] Kumarswamy R., Volkmann I., Thum T. (2014). Regulation and function of miRNA-21 in health and disease. RNA Biol..

[8] Guo X.Z., Ye X.L., Xiao W.Z., Wei X.N., You Q.H., Che X.H., Cai Y.J., Chen F., Yuan H., Liu X.J. (2015). Downregulation of VMP1 confers aggressive properties to colorectal cancer. Oncol. Rep..

[9] Ribas J., Ni X., Castanares M., Liu M.M., Esopi D., Yegnasubramanian S., Rodriguez R., Mendell J.T., Lupold S.E. (2012). A novel source for miR-21 expression through the alternative polyadenylation of VMP1 gene transcripts. Nucleic Acids Res..

[10] Moore L.M., Zhang W. (2010). Targeting miR-21 in glioma: a small RNA with big potential. Expert Opin. Ther. Targets.

[11] Davis S., Lollo B., Freier S., Esau C. (2006). Improved targeting of miRNA with antisense oligonucleotides. Nucleic Acids Res..

[12] Ebert M.S., Sharp P.A. (2012). Roles for microRNAs in conferring robustness to biological processes. Cell.

[13] Kluiver J., Gibcus J.H., Hettinga C., Adema A., Richter M.K., Halsema N., Slezak-Prochazka I., Ding Y., Kroesen B.J., van den Berg A. (2012). Rapid generation of microRNA sponges for microRNA inhibition. PLoS One.

[14] Chang H., Yi B., Ma R., Zhang X., Zhao H., Xi Y. (2016). CRISPR/cas9, a novel genomic tool to knock down microRNA in vitro and in vivo. Sci. Rep..

[15] Aloizou A.M., Pateraki G., Siokas V., Mentis A.A., Liampas I., Lazopoulos G., Kovatsi L., Mitsias P.D., Bogdanos D.P., Paterakis K. (2020). The role of MiRNA-21 in gliomas: hope for a novel therapeutic intervention?. Toxicol. Rep..

[16] Sathyan P., Zinn P.O., Marisetty A.L., Liu B., Kamal M.M., Singh S.K., Bady P., Lu L., Wani K.M., Veo B.L. (2015). Mir-21-Sox2 axis delineates glioblastoma subtypes with prognostic impact. J. Neurosci..

[17] Akers J.C., Ramakrishnan V., Kim R., Phillips S., Kaimal V., Mao Y., Hua W., Yang I., Fu C.C., Nolan J. (2015). miRNA contents of cerebrospinal fluid extracellular vesicles in glioblastoma patients. J. Neurooncol..

[18] Shi R., Wang P.Y., Li X.Y., Chen J.X., Li Y., Zhang X.Z., Zhang C.G., Jiang T., Li W.B., Ding W. (2015). Exosomal levels of miRNA-21 from cerebrospinal fluids associated with poor prognosis and tumor recurrence of glioma patients. Oncotarget.

[19] Clement K., Rees H., Canver M.C., Gehrke J.M., Farouni R., Hsu J.Y., Cole M.A., Liu D.R., Joung J.K., Bauer D.E. (2019). CRISPResso2 provides accurate and rapid genome editing sequence analysis. Nat. Biotechnol..

[20] Ma X., Kumar M., Choudhury S.N., Becker Buscaglia L.E., Barker J.R., Kanakamedala K., Liu M.F., Li Y. (2011). Loss of the miR-21 allele elevates the expression of its target genes and reduces tumorigenesis. Proc. Natl. Acad. Sci. U S A.

[21] Abels E.R., Maas S.L.N., Nieland L., Wei Z., Cheah P.S., Tai E., Kolsteeg C.-J., Dusoswa S.A., Ting D.T., Hickman S. (2019). Glioblastoma-associated microglia Reprogramming is mediated by functional transfer of extracellular miR-21. Cell Rep..

[22] Pfeffer S.R., Yang C.H., Pfeffer L.M. (2015). The role of miR-21 in cancer. Drug Dev. Res..

[23] Zhang B., Pan X., Cobb G.P., Anderson T.A. (2007). microRNAs as oncogenes and tumor suppressors. Dev. Biol..

[24] Krichevsky A.M., Gabriely G. (2009). miR-21: a small multi-faceted RNA. J. Cell Mol. Med..

[25] Shen T., Huang S. (2012). The role of Cdc25A in the regulation of cell proliferation and apoptosis. Anticancer Agents Med. Chem..

[26] Galaktionov K., Lee A.K., Eckstein J., Draetta G., Meckler J., Loda M., Beach D. (1995). CDC25 phosphatases as potential human oncogenes. Science.

[27] Li Y., Zhao S., Zhen Y., Li Q., Teng L., Asai A., Kawamoto K. (2011). A miR-21 inhibitor enhances apoptosis and reduces G(2)-M accumulation induced by ionizing radiation in human glioblastoma U251 cells. Brain Tumor Pathol..

[28] Wang P., Zou F., Zhang X., Li H., Dulak A., Tomko R.J., Lazo J.S., Wang Z., Zhang L., Yu J. (2009). microRNA-21 negatively regulates Cdc25A and cell cycle progression in colon cancer cells. Cancer Res..

[29] Chou S.T., Yen Y.C., Lee C.M., Chen M.S. (2010). Pro-apoptotic role of Cdc25A: activation of cyclin B1/Cdc2 by the Cdc25A C-terminal domain. J. Biol. Chem..

[30] Mazars A., Fernandez-Vidal A., Mondesert O., Lorenzo C., Prevost G., Ducommun B., Payrastre B., Racaud-Sultan C., Manenti S. (2009). A caspase-dependent cleavage of CDC25A generates an active fragment activating cyclin-dependent kinase 2 during apoptosis. Cell Death Differ..

[31] Leisser C., Rosenberger G., Maier S., Fuhrmann G., Grusch M., Strasser S., Huettenbrenner S., Fassl S., Polgar D., Krieger S. (2004). Subcellular localisation of Cdc25A determines cell fate. Cell Death Differ..

[32] Gary J.M., Simmons J.K., Xu J., Zhang S., Peat T.J., Watson N., Gamache B.J., Zhang K., Kovalchuk A.L., Michalowski A.M. (2020). Hypomorphic mTOR downregulates CDK6 and delays thymic pre-T LBL tumorigenesis. Mol. Cancer Ther..

[33] Bellail A.C., Olson J.J., Hao C. (2014). SUMO1 modification stabilizes CDK6 protein and drives the cell cycle and glioblastoma progression. Nat. Commun..

[34] Ojala P.M., Yamamoto K., Castanos-Velez E., Biberfeld P., Korsmeyer S.J., Makela T.P. (2000). The apoptotic v-cyclin-CDK6 complex phosphorylates and inactivates Bcl-2. Nat. Cell Biol..

[35] Orso F., Balzac F., Marino M., Lembo A., Retta S.F., Taverna D. (2013). miR-21 coordinates tumor growth and modulates KRIT1 levels. Biochem. Biophys. Res. Commun..

[36] Enderlin M., Kleinmann E.V., Struyf S., Buracchi C., Vecchi A., Kinscherf R., Kiessling F., Paschek S., Sozzani S., Rommelaere J. (2009). TNF-alpha and the IFN-gamma-inducible protein 10 (IP-10/CXCL-10) delivered by parvoviral vectors act in synergy to induce antitumor effects in mouse glioblastoma. Cancer Gene Ther..

[37] Liu M., Guo S., Stiles J.K. (2011). The emerging role of CXCL10 in cancer (Review). Oncol. Lett..

[38] He Z., Long J., Yang C., Gong B., Cheng M., Wang Q., Tang J. (2020). LncRNA DGCR5 plays a tumor-suppressive role in glioma via the miR-21/Smad7 and miR-23a/PTEN axes. Aging (Albany NY).

[39] Cao Q., Li Y.Y., He W.F., Zhang Z.Z., Zhou Q., Liu X., Shen Y., Huang T.T. (2013). Interplay between microRNAs and the STAT3 signaling pathway in human cancers. Physiol. Genomics.

[40] Ren X., Duan L., He Q., Zhang Z., Zhou Y., Wu D., Pan J., Pei D., Ding K. (2010). Identification of niclosamide as a new small-molecule inhibitor of the STAT3 signaling pathway. ACS Med. Chem. Lett..

[41] Bian Z., Ji W., Xu B., Huo Z., Huang H., Huang J., Jiao J., Shao J., Zhang X. (2021). Noncoding RNAs involved in the STAT3 pathway in glioma. Cancer Cell Int.

[42] Wang Y.Y., Sun G., Luo H., Wang X.F., Lan F.M., Yue X., Fu L.S., Pu P.Y., Kang C.S., Liu N. (2012). MiR-21 modulates hTERT through a STAT3-dependent manner on glioblastoma cell growth. CNS Neurosci. Ther..

[43] Wu W. (2018). MicroRNA, noise, and gene expression regulation. Methods Mol. Biol..

[44] Trohatou O., Zagoura D., Bitsika V., Pappa K.I., Antsaklis A., Anagnou N.P., Roubelakis M.G. (2014). Sox2 suppression by miR-21 governs human mesenchymal stem cell properties. Stem Cells Transl. Med..

[45] Ni Y., Zhang K., Liu X., Yang T., Wang B., Fu L., A L., Zhou Y. (2014). miR-21 promotes the differentiation of hair follicle-derived neural crest stem cells into Schwann cells. Neural Regen. Res..

[46] Singh S.K., Marisetty A., Sathyan P., Kagalwala M., Zhao Z., Majumder S. (2015). REST-miR-21-SOX2 axis maintains pluripotency in E14Tg2a.4 embryonic stem cells. Stem Cell Res..

[47] Annovazzi L., Mellai M., Caldera V., Valente G., Schiffer D. (2011). SOX2 expression and amplification in gliomas and glioma cell lines. Cancer Genomics Proteomics.

[48] Schmitz M., Temme A., Senner V., Ebner R., Schwind S., Stevanovic S., Wehner R., Schackert G., Schackert H.K., Fussel M. (2007). Identification of SOX2 as a novel glioma-associated antigen and potential target for T cell-based immunotherapy. Br. J. Cancer.

[49] Chen Y., Liu W., Chao T., Zhang Y., Yan X., Gong Y., Qiang B., Yuan J., Sun M., Peng X. (2008). MicroRNA-21 down-regulates the expression of tumor suppressor PDCD4 in human glioblastoma cell T98G. Cancer Lett..

[50] Mei M., Ren Y., Zhou X., Yuan X.B., Han L., Wang G.X., Jia Z., Pu P.Y., Kang C.S., Yao Z. (2010). Downregulation of miR-21 enhances chemotherapeutic effect of taxol in breast carcinoma cells. Technol. Cancer Res. Treat.

[51] Li T., Li R.S., Li Y.H., Zhong S., Chen Y.Y., Zhang C.M., Hu M.M., Shen Z.J. (2012). miR-21 as an independent biochemical recurrence predictor and potential therapeutic target for prostate cancer. J. Urol..

[52] Sicard F., Gayral M., Lulka H., Buscail L., Cordelier P. (2013). Targeting miR-21 for the therapy of pancreatic cancer. Mol. Ther..

[53] Arya D., Sachithanandan S.P., Ross C., Palakodeti D., Li S., Krishna S. (2017). MiRNA182 regulates percentage of myeloid and erythroid cells in chronic myeloid leukemia. Cell Death Dis..

[54] Li X., Chen W., Zeng W., Wan C., Duan S., Jiang S. (2017). microRNA-137 promotes apoptosis in ovarian cancer cells via the regulation of XIAP. Br. J. Cancer.

[55] Yoshino H., Yonemori M., Miyamoto K., Tatarano S., Kofuji S., Nohata N., Nakagawa M., Enokida H. (2017). microRNA-210-3p depletion by CRISPR/Cas9 promoted tumorigenesis through revival of TWIST1 in renal cell carcinoma. Oncotarget.

[56] Zhou S.J., Deng Y.L., Liang H.F., Jaoude J.C., Liu F.Y. (2017). Hepatitis B virus X protein promotes CREB-mediated activation of miR-3188 and Notch signaling in hepatocellular carcinoma. Cell Death Differ..

[57] Ho T.T., Zhou N., Huang J., Koirala P., Xu M., Fung R., Wu F., Mo Y.Y. (2015). Targeting non-coding RNAs with the CRISPR/Cas9 system in human cell lines. Nucleic Acids Res..

[58] Huo W., Zhao G., Yin J., Ouyang X., Wang Y., Yang C., Wang B., Dong P., Wang Z., Watari H. (2017). Lentiviral CRISPR/Cas9 vector mediated miR-21 gene editing inhibits the epithelial to mesenchymal transition in ovarian cancer cells. J. Cancer.

[59] Bautista-Sanchez D., Arriaga-Canon C., Pedroza-Torres A., De La Rosa-Velazquez I.A., Gonzalez-Barrios R., Contreras-Espinosa L., Montiel-Manriquez R., Castro-Hernandez C., Fragoso-Ontiveros V., Alvarez-Gomez R.M. (2020). The promising role of miR-21 as a cancer biomarker and its importance in RNA-based therapeutics. Mol. Ther. Nucleic Acids.

[60] Hanna J., Hossain G.S., Kocerha J. (2019). The potential for microRNA therapeutics and clinical research. Front. Genet..

[61] Li C., Zamore P.D. (2018). Preparation of antisense oligonucleotides to inhibit miRNA function. Cold Spring Harb. Protoc..

[62] Corsten M.F., Miranda R., Kasmieh R., Krichevsky A.M., Weissleder R., Shah K. (2007). MicroRNA-21 knockdown disrupts glioma growth in vivo and displays synergistic cytotoxicity with neural precursor cell delivered S-TRAIL in human gliomas. Cancer Res..

[63] Krutzfeldt J., Kuwajima S., Braich R., Rajeev K.G., Pena J., Tuschl T., Manoharan M., Stoffel M. (2007). Specificity, duplex degradation and subcellular localization of antagomirs. Nucleic Acids Res..

[64] Maguire C.A., Gianni D., Meijer D.H., Shaket L.A., Wakimoto H., Rabkin S.D., Gao G., Sena-Esteves M. (2010). Directed evolution of adeno-associated virus for glioma cell transduction. J. Neurooncol..

[65] Wang D., Zhang F., Gao G. (2020). CRISPR-based therapeutic genome editing: strategies and *in vivo* delivery by AAV vectors. Cell.

[66] Cota-Coronado A., Diaz-Martinez N.F., Padilla-Camberos E., Diaz-Martinez N.E. (2019). Editing the central nervous system through CRISPR/Cas9 systems. Front. Mol. Neurosci..

[67] El Fatimy R., Subramanian S., Uhlmann E.J., Krichevsky A.M. (2017). Genome editing reveals glioblastoma addiction to microRNA-10b. Mol. Ther..

[68] Chen H.C. (2005). Boyden chamber assay. Methods Mol. Biol..

[69] Franken N.A., Rodermond H.M., Stap J., Haveman J., van Bree C. (2006). Clonogenic assay of cells in vitro. Nat. Protoc..

[70] Love M.I., Anders S., Kim V., Huber W. (2015). RNA-Seq workflow: gene-level exploratory analysis and differential expression. F1000Res..

[71] Maas S. (2020). Glioblastoma hijacks microglial gene expression to support tumor growth. Journal of Neuroinflammation.

