# Supplementary material for: CRISPR-Cas knockout of miR21 reduces glioma growth
Source: Mol Ther Oncolytics. 2022 Apr 6;25:121–36. doi: 10.1016/j.omto.2022.04.001 (PMC9052041; doi:10.1016/j.omto.2022.04.001)
Supplement: Document S1. Figures S1–S7; Tables S1 and S2 [file mmc1.pdf]

OMTO, Volume *25*

Supplemental information

CRISPR-Cas knockout of miR21 reduces glioma growth

Lisa Nieland, Thomas S. van Solinge, Pike See Cheah, Liza M. Morsett, Joseph El Khoury, Joseph I. Rissman, Benjamin P. Kleinstiver, Marike L.D. Broekman, Xandra O. Breakefield, and Erik R. Abels

SUPPLEMENTAL INFORMATION

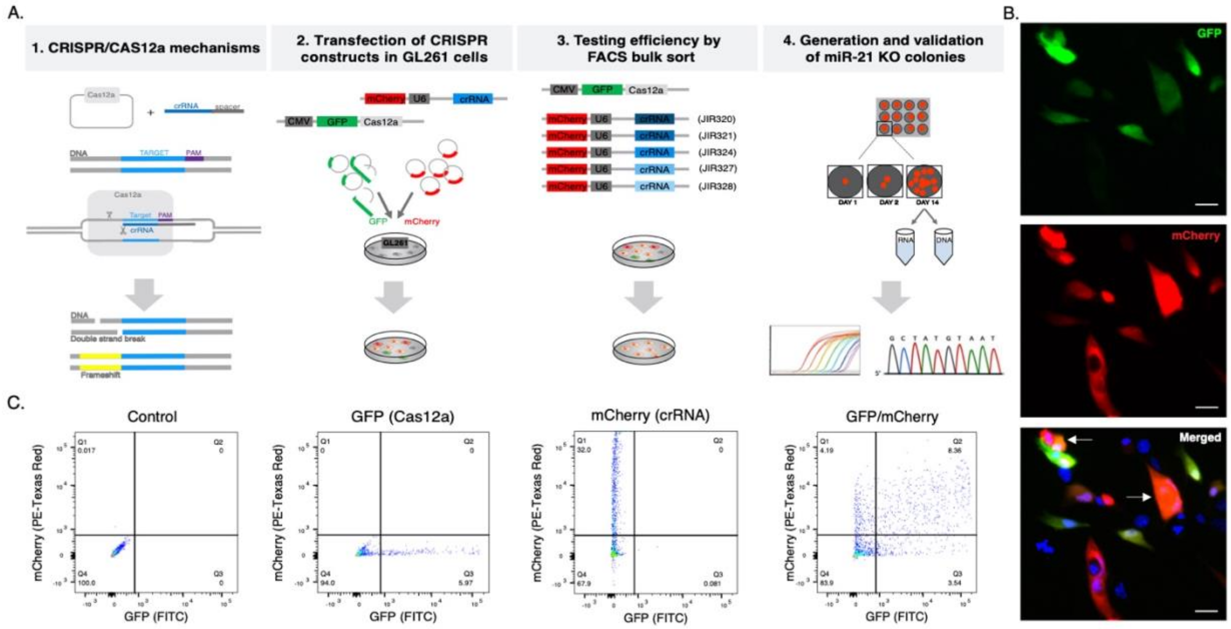

Figure S1. CRISPR-Cas12 targeting the miR-21 allele. (**A-1**) Schematic overview of gene editing mechanism used; endonuclease Cas12a and sgRNA with a crRNA specific for a PAM on the miR-21 allele. Both Cas12a protein and sgRNA form a ribonucleoprotein complex. If the Cas12a-crRNA complex successfully binds the DNA, a DNA cleavage downstream of the target DNA will occur. This double strand break will be repaired by the non-homologous end joining causing INDELs in the DNA. These INDELs lead to a disruption of sequences, creating loss of function. (**A-2**) CRISPR components are expressed following transfection of DNA plasmids into the mouse glioma cell line. To monitor transfection efficiency CRISPR plasmids are also encoded for GFP (Cas12a expressing construct) or mCherry (crRNA expressing construct). Efficiency and co-expression of GFP (Cas12a) and mCherry (crRNA) was tracked by fluorescence microscopy and FACS. (**A-3**) Five sgRNAs (each containing a different crRNA sequence) were specifically designed to target the miR-21 sequence within the *Vmp1* gene. Co-expression of the Cas12a

plasmid with each specific crRNA was tested in parallel. Upon successful double transfection of GL261 cells, double positive cells will appear orange. (**A-4**) To generate a clonal cell line, cells were transfected with Cas12a and crRNA expression plasmids following single cell sorting in 96-well plates. Both RNA and DNA were extracted after stable colonies were grown in sufficient amounts. Validation of fully functional CRISPR-Cas12a activity by the presence of disruption in miR-21 sequence was confirmed by sequence analysis and validated by expression level by qPCR. (**B**) Co-transfection of GL261 cells were analyzed using fluorescent microscopy. Single fluorescent channels show GFP (green) only and mCherry (red) only. Merged images show cells co-expressing both CRISPR plasmids (orange; white arrows). Scale bar 10 μm. (**C**) Representative FACS plots showing expression of crRNA by measurement of mCherry on the y-axis and Cas12a by GFP on the x-axis. From left to right: control: cells neither positive for GFP or mCherry; cells positive for GFP; cells positive for mCherry; and cells co-expressing mCherry and GFP.

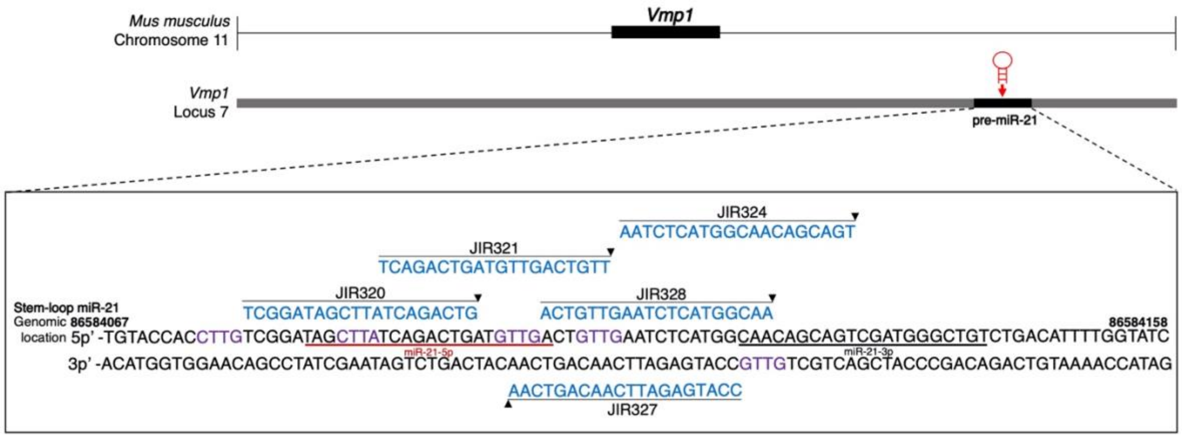

Figure S2. Targeting the miR-21 sequence by using CRISPR. *Mus musculus* miR-21 stem-loop 92 nt (genomic location: 86585067-86584158) illustrated within the 3' UTR of the *Vmp1* gene on chromosome 11. The miR-21 stem-loop contains both miR-21-5p 22 nt (5-TAGCTTATCAGACTGATGTTGA-3) marked with a red line and miR-21-3p 22 nt (5-CAACGCAGTCGATGGGCTGT-3) marked with a black line. Multiple sgRNAs were designed containing specific crRNAs (blue) recognizing different PAMs (purple) at various locations in the miR-21 stem-loop sequence. sgRNA JIR327 worked most efficiently, its crRNA recognizes a PAM site in the reverse sequence and cuts within the miR-21-5p.

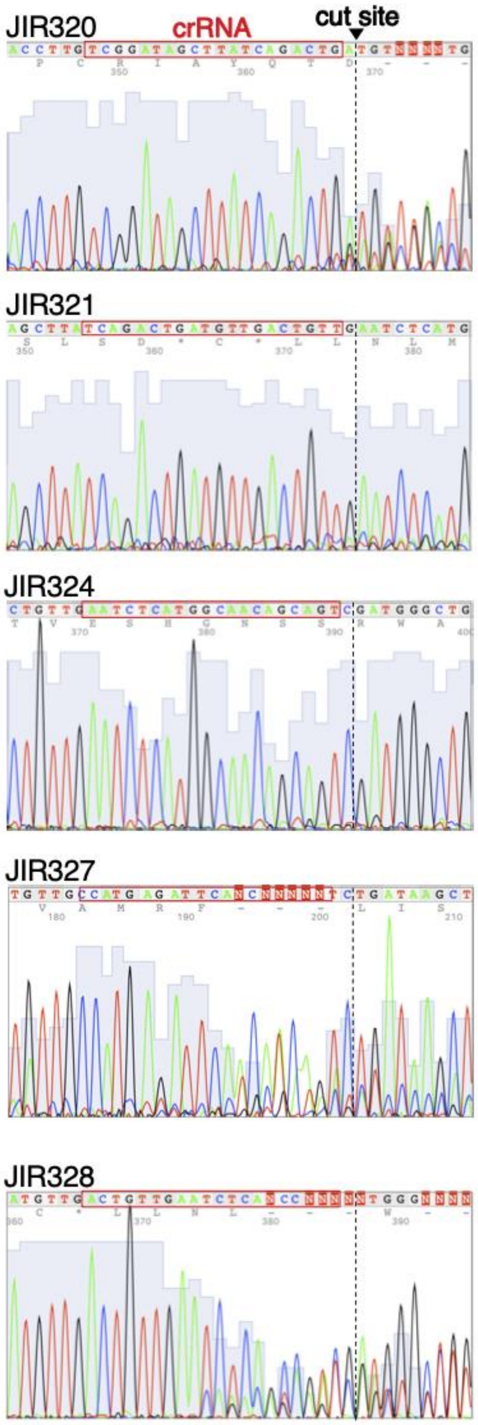

Figure S3. Sanger sequencing results of 5 tested crRNAs. The effectiveness of 5 specific crRNAs (JIR320; JIR321; JIR324; JIR327 and JIR328) was analyzed by Sanger sequencing. The

crRNA sequences are shown in a red box, and the cut site is displayed as a dotted line. Plasmid JIR324 showed no CRISPR activity and was therefore eliminated.

A.

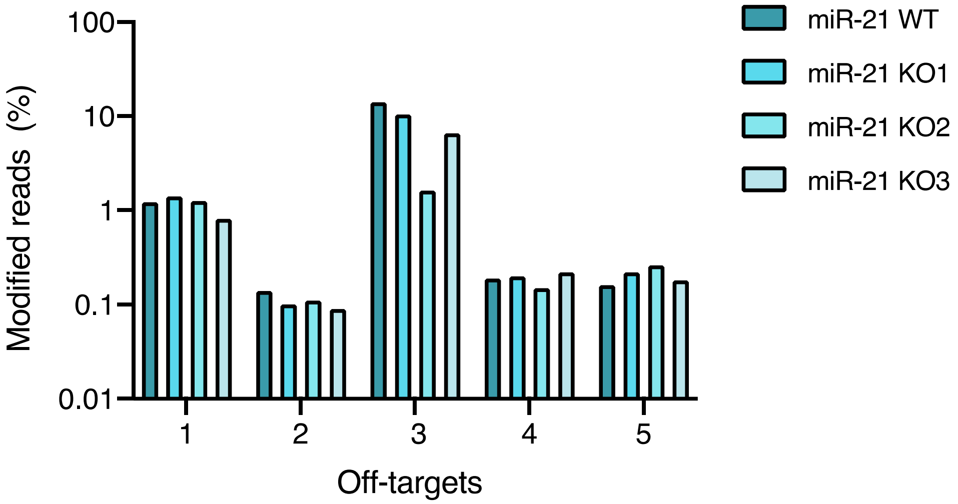

Figure S4. Off-target effects of the generated miR-21 KO clones. The program CRISPResso2 was used to analyze the off-target NGS data. The percentages of the modified reads are displayed on the log scale y-axis comparing WT to the miR-21 KO colonies 1-3 for the top five off-targets (**Supplementary Table S2**). The modification rates were not significantly different compared to WT.

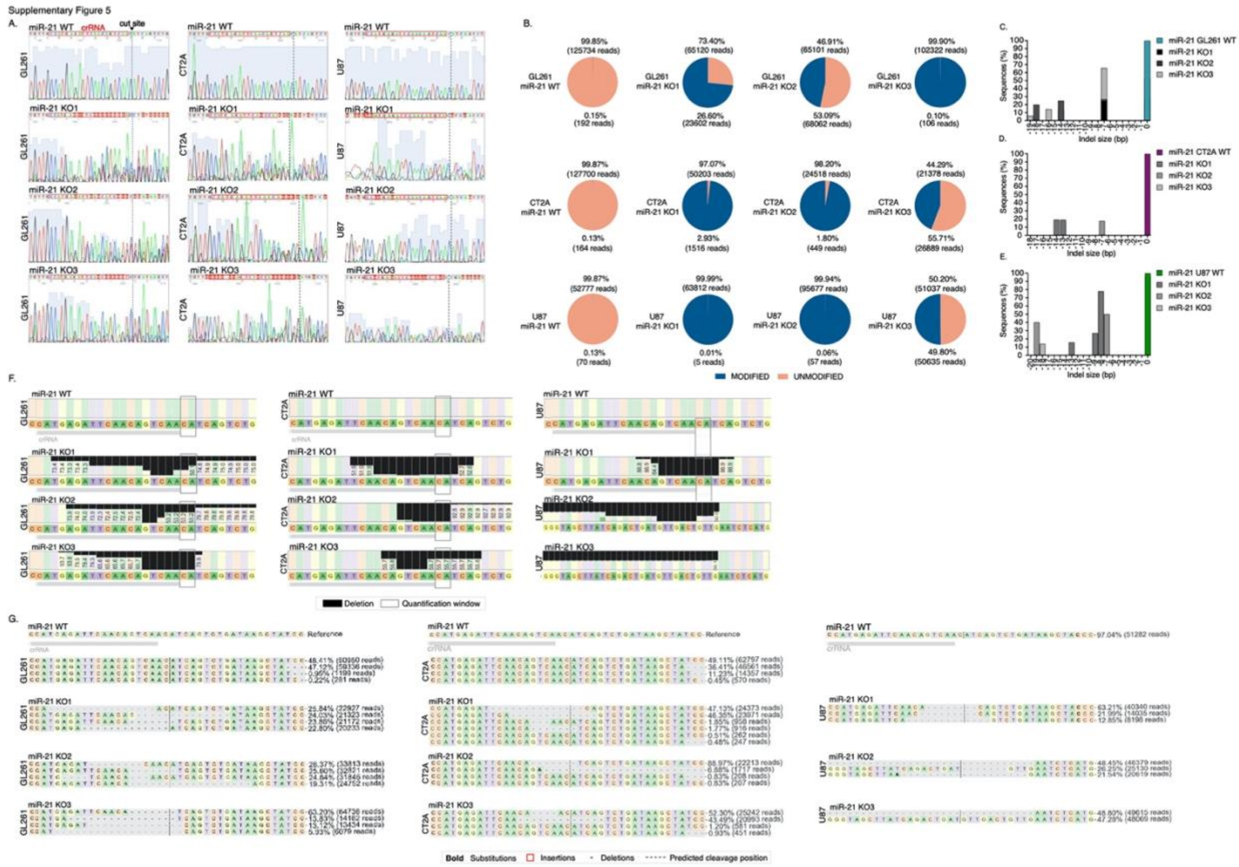

Figure S5. miR-21 KO GL261 and CT2A clones generated by CRISPR-Cas12a. (**A**) The CRISPR edited clones KO 1-3 were aligned to the GL261 WT, the CT2A WT and the U87 WT sequence to analyze the CRISPR induced insertion-deletion (INDEL). (**B**) GL261 miR-21 KO colonies 1-3 had 66,120 (73.40%), 65,101 (46.91%), 102,322 (99.90%) modified reads. CT2A miR-21 KO colonies 1-3 had 50,203 (97.07%), 24,518 (98.20%), 21,378 (44.29%) modified reads. U87 miR-21 KO colonies 1-3 had 63,812 (99.99%), 95,677 (98.294%), 51,037 (50.20%) modified reads. (**C**) The INDEL sizes varied but the most common INDEL was a seven base pair deletion in GL261 miR-21 KO clones 1-3. GL261 WT had no base pair deletions (99.99%). (**D**) The CT2A miR-21 KO clones 1-3 had a 7, 13 or 14 base pair deletion. CT2A WT had no base pair deletions (99.99%). (**E**) The U87 miR-21 KO clones 1-3 had a 7, 8 or 9 or >18 base pair deletion. U87 WT had no base pair deletions (99.99%). (**F**) NGS showed quantification of nucleotide percentages

and deletions (black squares) for GL261, CT2A and U87 miR-21 KO clones 1-3. The alignment also includes reads with INDELs outside the quantification window. The crRNA target sequence is displayed in a grey solid line. (**G**) INDEL quantification represents percentages of total reads of individual INDELs within miR-21 KO clones 1-3 of GL261, CT2A and U87 lines aligned to their WT reference allele.

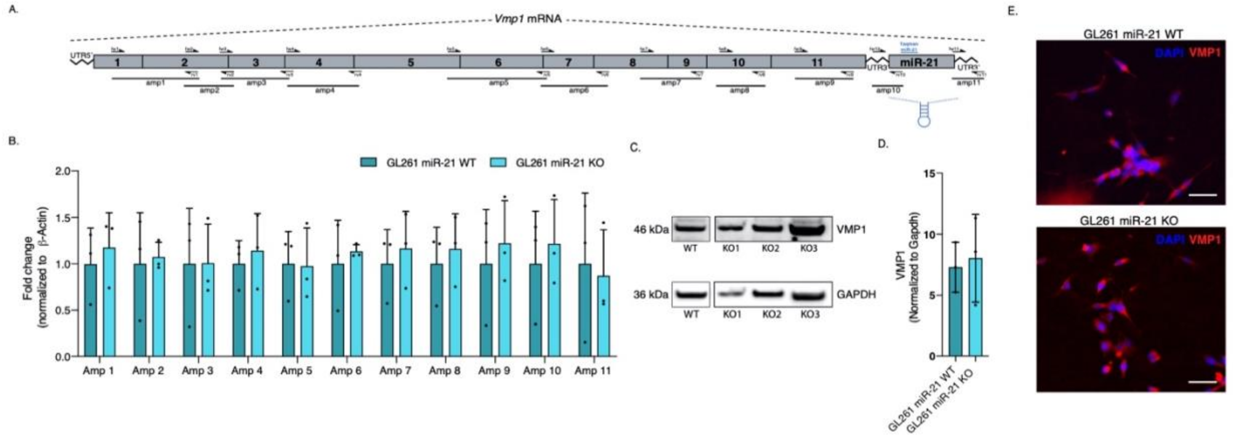

Figure S6: miR-21 KO does not affect Vmp1 expression. (**A**) Locations of 11 different specifically designed primer sets covering the *Vmp1* mRNA. Each spans one of the 11 exons sequentially and their amplicons are displayed with a black line. The miR-21 sequence is shown between two untranslated regions (UTR) after exon 11 at the 3'end of the *Vmp1* mRNA sequence. The location within the miR-21 sequence which is targeted by the Taqman primer miR-21-5p is colored in blue. (**B**) Primers aligning the *Vmp1* sequence at different locations show similar CT values for GL261 WT compared to GL261 miR-21 KO3. CT values were normalized to ß-Actin and reactions were done in triplicate. (**C**) By western blot analysis GL261 WT and GL261 miR-21 KO clones 1-3 showed a band at 46 kDa, corresponding to the molecular weight for VMP1 compared to GAPDH, 36 kDa, using antibodies specific for each protein. (**D**) GL261 WT and GL261 miR-21 KO clones 1-3 show similar levels of VMP1 normalized to GAPDH. Data represent three independent experiments and are presented as the mean with SEM (error bars), unpaired t test. (**E**) Both GL261 WT and GL261 miR-21 KO cells stained positively for VMP1 (red). DAPI (blue) was used to stain the nucleus. Scale bar 10 µm.

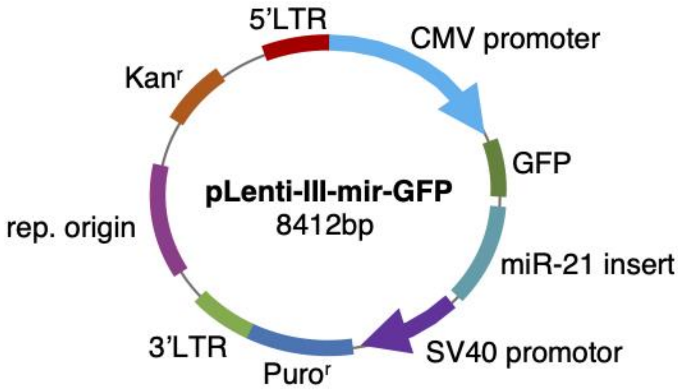

Figure S7: Lentiviral vector miR-21. Illustration shows the LentimiRa-GFP-mmu-mir-21 vector (mm10221, abm) map co-expressing the inserted miR-21 (517 bp) sequence and GFP under a CMV promoter.

Table S1: Five primer sets flanking the top 5 off-targets.

Off-target	Primer number	Primer sequence	Amplicon size	Amplicon location
1	Fw1	5-ATGGCTGGTTTTGTGTCAACT-3	224	20582247-20582267
1	Rv1	5-ATGGGAGCTGGCTTGTTTCC-3		20582471-20582452
2	Fw2	5-GCTGGGGATTCAGTTGCTAGT-3	165	75786447-75786467
2	Rv2	5-TGGGGAGAGGGAAAGAGAGTT-3		75786611-75786591
3	Fw3	5-TGTGTGGGGCTACATCAACC-3	188	71697072-71697091
3	Rv3	5-CCTTGAAGCATTTCCCAACTGT-3		71697259-71697238
4	Fw4	5-TTTTGCCACACCAGTGAGAA-3	124	36318288-36318307
4	Rv4	5-ACACAAAGAAAACATGGACATTGA-3		36318411-36318388
5	Fw5	5-AGGGAAGTACCACATAAGCGTT-3	317	100795709-100795730
5	Rv5	5-TCTACCTCCACTGATCACCGA-3		100796025-100796005

Table S2: Overview of the top 5 off-targets.

Off-target	DNA	Location	Position	Direction	Mismatches
crRNA	5-TTGCCATGAGATTCAACAGTCAA-3	chr11	86584100	+	0
1	5-TTTCCATGAGAT**c**CAACAGT**a**AA-3	chr16	20582317	+	2
2	5-TTAC**a**ATGAGAT**a**CAACAGT**t**AA-3	chr17	75786546	-	3
3	5-TTACCA**a**GAGAT**ca**AACAGTCAA-3	chr7	71697171	-	3
4	5-TTACCATGAGA**c**TC**c**AC**t**GTCAA-3	chr6	36318362	-	3
5	5-TTACCATGA**t**ATTCAACAG**a**CA**t**-3	chr9	100795836	+	3
